# A Method Framework for Automatic Airspace Reconfiguration-Monte Carlo Method for Eliminating Irregular Sector Shapes Generated by Region Growth Method

**DOI:** 10.3390/s19183934

**Published:** 2019-09-12

**Authors:** Zhijian Ye, Fanhe Kong, Baocheng Zhang, Wei Gao, Jianfeng Mao

**Affiliations:** 1 College of Air Traffic Management, Civil Aviation University of China, Tianjin 300300, China; 2 ATC Dep, Dalian ATM Station of Civil Aviation of China, Dalian 116033, China; 3 School of Science and Engineering, Shenzhen Key Laboratory of IoT Intelligent Systems and Wireless Network Technology, The Chinese University of Hong Kong, Shenzhen 518172, China

**Keywords:** airspace reconfiguration, irregular boundary smoothing, dynamic Monte Carlo method by changing location of flexible vertices, Monte Carlo method by radius changing, Voronoi diagram, graph cutting, multi-objective optimization

## Abstract

With the growth of air traffic demand in busy airspace, there is an urgent need for airspace sectorization to increase air traffic throughput and ease the pressure on controllers. The purpose of this paper is to develop a method framework that can perform airspace sectorization automatically, reasonably, which can be used as an advisory tool for controllers as an automatic system, especially for eliminating irregular sector shapes generated by simulated annealing algorithm (*SAA*) based on the region growth method. The two graph cutting method, dynamic Monte Carlo method by changing location of flexible vertices (MC-CLFV) and Monte Carlo method by radius changing (MC-RC) were developed to eliminate irregular sector shapes generated by SAA in post-processing. The experimental results show that the proposed method framework of airspace sectorization (AS) can automatically and reasonably generate sector design schemes that meet the design criteria. Our methodology framework and software can provide assistant design and analysis tools for airspace planners to design airspace, improve the reliability and efficiency of airspace design, and reduce the burden of airspace planners. In addition, this lays the foundation for reconstructing airspace with the more intelligent method.

## 1. Introduction

**Figure 1:**
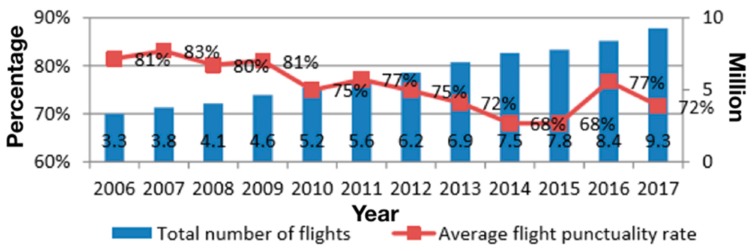
Average flight punctuality rate and total number of flights.

In recent years, the air traffic flow of China has been increasing at an average annual rate of 11 percent, and air transport is developing rapidly. With the rapid development of national traffic volume, China’s airports and airspace system are under increasing pressure, which leads to frequent flight delays. In 2010, the fixed assets investment of Chinese airports increased from 44.150 billion Yuan to 56.08 billion Yuan, with a compound annual growth rate of 2.54% [1]. Thus in order to solve the imbalance between demand and capacity, specific measures include strengthening the construction of infrastructure and expanding fleet was taken. However, according to the statistics of CAAC (Civil Aviation Administration of China), the average flight punctuality rate in China maintained at a low level (as shown in Figure 1) in the past few years [2].

This indicates that the government needs to not only strengthen the construction of infrastructure, but also give advice on how to manage the airspace effectively. The current Chinese National Airspace architecture is reaching the ability limits to accommodate the increases in traffic demand. As the key limiting factor, today’s sector boundaries are largely determined by historical user profiles, which have evolved slowly over time. Consequently the sector geometry has stayed relatively constant despite the fact that route structures and demand have changed dramatically over the years. There are usually two forms of traffic flow growth in airspace. The first is that the traffic flow of different routes has different growth rates with time. Some sectors have excessive task load, while others have a relatively small task load. In this case, the problem that throughput is limited by sector capacity can be solved by adjusting sector structure and keeping the number of sectors unchanged. The second is that the traffic flow of all routes in the whole airspace has increased significantly. The control workload cannot be effectively reduced by just adjusting sector structure without airspace sectorization (AS) [3,4]. 

Although AS is the most potential method to increase throughput of airspace as well as to better use scarce resources, there is a serious challenge to realize it. Except for the accessibility of airspace sectorization by air traffic controller, a lot of constraints must be considered in accordance with the demand of air traffic control operation. Since AS needs to consider the constraints of the balance of task load, shape, connectivity and compactness, which are beyond the limits of human ability, a mathematical optimization tool is needed to support this process [5,6].

In terms of problem solving technology, AS is essentially a special partitioning technology. Partitioning technology has been widely used in the analysis and design of various agricultural [7], computer [8,9], communication [10,11], economic [12], sea [13], road [14], intermodal transport [15], power [16] and other partitionable systems and networks. AS and these partitioning problems have similarities in load distribution, but it makes AS different from these partitioning problems that coordination workload varies with relative position relationship between AS boundary and routes.

A lot of AS research has been carried out. Delahaye, et al. [17] initiated the study of the balanced sector of airspace in order to increase air traffic control capacity in high density areas. Genetic algorithms were used to compute a balanced regrouping of air traffic control sectors thus to optimally reduce the number of controller teams [18]. A decision support tool based on fuzzy logic was proposed to determine the number of open sectors during a given time period [19]. Related researches include dynamic airspace configuration (DAC), dynamic airspace sectorization (DAS) and airspace configuration. In DAC, the airspace is adjusted in real-time to accommodate fluctuating demand patterns [5,20,21,22,23,24]. A detailed overview of recent work of DAC is given by Zou, et al. [25]. Transitional workload is considered in most of these DAC models. Gerdes, Temme and Schultz [23] clearly distinguished DAC from DAS. DAS is a flexible method, encompassing the idea of unstructured and (rigid) structured airspace, and differing from DAC, where predefined airspace blocks are combined to form new structures (merging and splitting). DAS approach creates a less familiar setting for controllers but can be used for situations where no basic knowledge concerning the best airspace structure is available. This paper mainly solves the problem of increasing the number of sectors or improving sectors caused by the rapid growth of China’s traffic volume. Our objective is to adjust the sector structure to increase capacity. Civil Aviation of China stipulates that as long as the sector structure changes, controllers must carry out pre-job training. The problem of transitional workload is another problem of controller’s assignment with minimizing training cost. AS in this paper is closer to DAS, because it is a strategic or tactical AS before the implementation of AS scheme.

Flener and Pearson [26] systematically surveyed the algorithmic aspects of methods for automatic airspace sectorization, for an intended readership of experts on air traffic management. Most of the airspace sectorization models were based on two approaches. The first one is the graph-based model. In this type of model, a graph is constructed whose vertices represent the intersections of the existing trajectories while edges represent segments [14,17,27,28,29]. Several recent published papers also use this type of model [25,30,31]. The graph-based model could use some mature technology in graph partition, such as the MIN-CUT graph partition used as minimizing the coordination task load. However, a graph partition does not define the sector boundaries, so actual sectors have to be constructed from the resulting vertex sets in a geometric post-processing step. The second one is the region-based model in which the airspace is initially partitioned into some type of regions, which are smaller than the studied sectors, so that the combinatorial problem of partitioning these regions in principle needs no geometric post-processing step [32,33,34,35,36,37,38,39,40]. Unfortunately, shapes of obtained sectors, which use the region-based model still have to need post-processing. Although the region-based model is essentially a down-top combination approach, in which workload among the sectors was balanced by maintaining the sector shape parameters within acceptable limits, but it made them operationally undesirable that there were some corners or Z-shape among these sectors even after post-processing [41,42].

Ref. [43,44] developed another top-down bi-partition approach to cut airspace into sectors. Xue [20], Gerdes, Temme and Schultz [23], Xue [45], Gerdes, et al. [46] also used top-down approach to redesign airspace sector while the generation of sector boundary was based on Voronoi diagrams. With the Voronoi diagram, the convexity requirement is automatically satisfied. The success of this approach depends heavily on the location of Voronoi generating points. For example, in airspace of 200 km × 200 km, if the alternative Voronoi generating points are distributed every 10 km, there will be 400 points. In the case of dividing the airspace into five sectors, the number of alternative locations at five production points is $400^{5}$. If this approach is applied to larger airspace and divided into more sectors, the computation time may be unacceptable. When estimating the coordinating task load, Xue [20] pointed out that the boundary-crossing of air traffic control center was neglected in his work. Obviously, this will lead to an unbalanced sector task load. If the sector generated by the algorithm is applied to the actual sector, the actual workload of the sector near the studying airspace boundary will be larger than the result of calculation. In addition, if all sectors are Voronoi diagrams, in some cases, it is difficult to find a suitable seed point for Voronoi cell (VC) generation to balance the workload and meet other constraints.

Delahaye, et al. [47] have proposed the use of floating-point chromosomes, and have developed special operators to maintain the integrity of the chromosome for use in a floating point representation, contending that the convergence rate is faster in the floating-point case. When the studying space is discretized by n VCs (Voronoi cells) and the number of sectors is k, the number of possible groupings will be $\vartheta_{n}^{k}$ as following.

(1) $$\vartheta_{n}^{k}=\frac{1}{n!}\sum_{i=0}^{i=n-1}{\left(-1\right)}^{i}\left(\frac{n!}{i!\left(n-i\right)!}\right){\left(n-i\right)}^{k}.$$

They pointed out that this kind of combination is a non-deterministic polynomial hard problem and that stochastic optimization is a good candidate to address it. Pawlak, et al. [48] also used this type of floating-point chromosomes to group cells by the finite-element method. Sergeeva, et al. [49] developed a more complex floating-point chromosome for 3D reconfiguration, which includes two parts, the first part represents coordinates of sector centers and the second part contains the associated vertical extension of each sector center. However, these chromosomes are hard to understand especially how they work in the genetic algorithm.

The reconfiguration problem is well-known for the NP-hard problem [47,50,51]. Simulated annealing (SA) is a well-studied local search and meta-heuristic used to address discrete and, to a lesser extent, continuous optimization problems. It is a probabilistic technique for approximating the global optimum of a given function. Specifically, it is a meta-heuristic to approximate global optimization in a large search space. It is often used when the search space is discrete. The key feature of simulated annealing is that it provides a mechanism to escape local optima by allowing hill-climbing moves (i.e., moves, which worsen the objective function value) in hopes of finding a global optimum [52]. Johnson, et al. [53], [54] is the pioneer who uses SA to graph coloring and number partitioning. Rahimian, et al. [55] proposed a fully distributed algorithm called JA-BE-JA, which uses local search and simulated annealing techniques for two types of graph partitioning: Edge-cut partitioning and vertex-cut partitioning. These partitioning algorithms have some similar objective, such as minimizing cut cost and balancing weights of vertexes. However, they still are different from AS, which require balancing the total workload (monitoring and coordinating task loads), minimizing the coordination task load, as well as thinking about dwell time, convex shape, connectivity and compactness.

In summary, the bottom-up method is prone to appear C-shaped, zigzag, trapezoidal and other irregular shapes, while the top-down method is prone to appear as a narrow triangle, which is not easy to ensure convexity. In addition, the method of a random Voronoi diagram is like looking for a needle in a haystack to get an optimal solution. It is doubtful that the multi-layer random Voronoi graph without boundary reprocessing not only guarantees the workload balance of the graph, but also guarantees the convexity of the sector. Therefore, we must design an innovative solution to the AS problem.

The aim of this paper is to establish a framework of solving methods including a series of multi-objective (balance the task load, minimize the task load imbalance, minimize the total coordinating task load, minimize the total coordinating task load, minimize the cost of short dwell time and minimize the cost of reentering the same sector) solving methods that can perform AS automatically at the strategically planning stage, while meeting the constraints of connectivity and compactness in optimal process, as well to smooth the boundary for an accessible shape of the sector. In order to improve the reliability of the design, we synthesized the region growth method and the graph cutting method. Firstly, we use the region growth method based on the Voronoi diagram to generate the initial optimal solution. Then, two graph cutting algorithms are used to eliminate irregular sector boundary caused by region growing algorithm.

This paper makes the following specific contributions:

1. Both of two graph cutting algorithms proposed by us can eliminate the irregular sector boundary caused by the region growing algorithm as well as other criteria can also be guaranteed.
2. The MC-CLFV algorithm based on flexible vertices generated deducted from the simulated annealing algorithm (SAA) result greatly simplifies the complexity of graphic cutting algorithm.
3. The MC-RC algorithm shows some advantages to reduce narrow blocks while the MC-CLFV algorithm is superior to the MC-RC algorithm for increasing traffic throughput.
4. We also found that appropriately allowing some non-convex boundaries is a measure to reduce the total workload, and blindly pursuing convex boundaries will increase the total workload.
5. The solution framework proposed in this paper improves the reliability of obtaining the optimal design scheme in airspace, and will reduce the design load and completion time of designers.

These studies are of great significance to improve air traffic throughput and maintain the task load of controllers at a reasonable level. This paper consists of five sections. Beginning with the introduction, Section 2 describes problem formulation. Section 3 elaborates the details of the solution method, which including SAA and the two post-processing method. Experimental results for a realistic scenario are reported in Section 4. Section 5 presents discussions. Conclusions and opportunities for further work are presented in Section 6.

## 2. Problem Formulation

### 2.1. Boundary and Route Network

**Figure 2:**
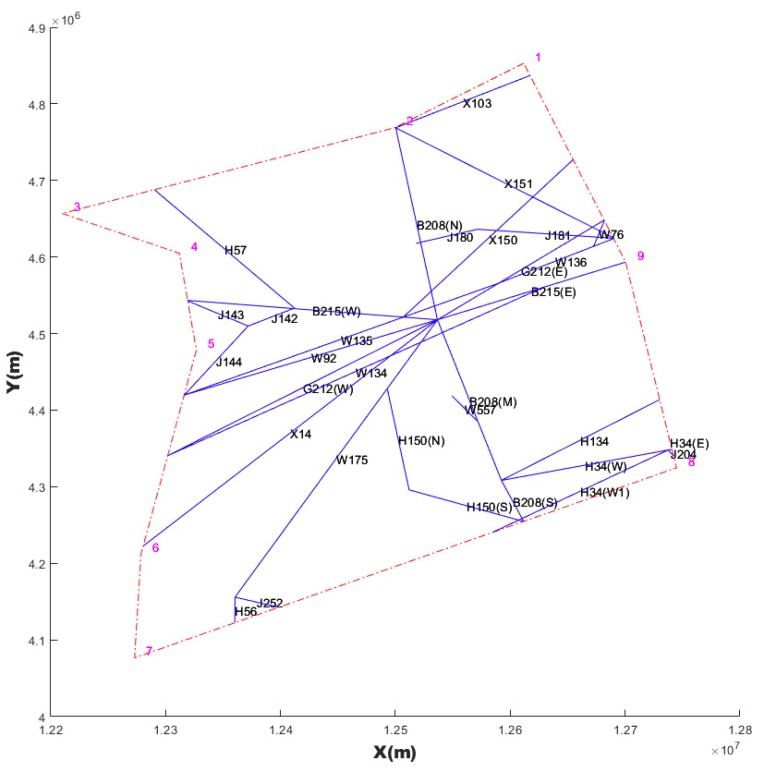
Route network and boundary in TYN airspace.

In order to clearly show the problem, we use Taiyuan Airspace (TYN, Taiyuan Airport is defined as TYN by the International Air Transport Association with three-character code) as the background in the following definitions of airspace structure. The boundary points are expressed as B. B is a n × 2 matrix, and is listed anti-clockwise. The boundary line is the red dotted line in Figure 2. Each row of matrix B represents a geographic coordinate of a point.

The route network consists of all segments (blue line in Figure 2) in airspace. Table 1 is an example of the route network shown in Figure 2. Due to the paper length limit, we listed only a part of the data. Despite having the same name, the segment is distinguished by the route course, such as B208 (N), B208 (M) and B208 (S). Each segment includes the coordinates of the starting and ending points and the daily traffic volume.

### 2.2. Voronoi Diagrams Generating Method

VCs are adopted to discretize the reconfiguration airspace in this paper. Given some generating points (seeds of the Voronoi diagram), the Voronoi diagram divides airspace into a group of convex polygons with no overlaps. In theory, the higher the number of generating points, the greater the solution space. Although the small granularity can result in more balanced sectors, it will take more time to calculate. The distribution of generating points in space also affects the solution. The following three methods were used to generate seeds of the Voronoi diagrams.

#### 2.1.1. Seeds Were Generated Randomly in the Studying Airspace

**Figure 3:**
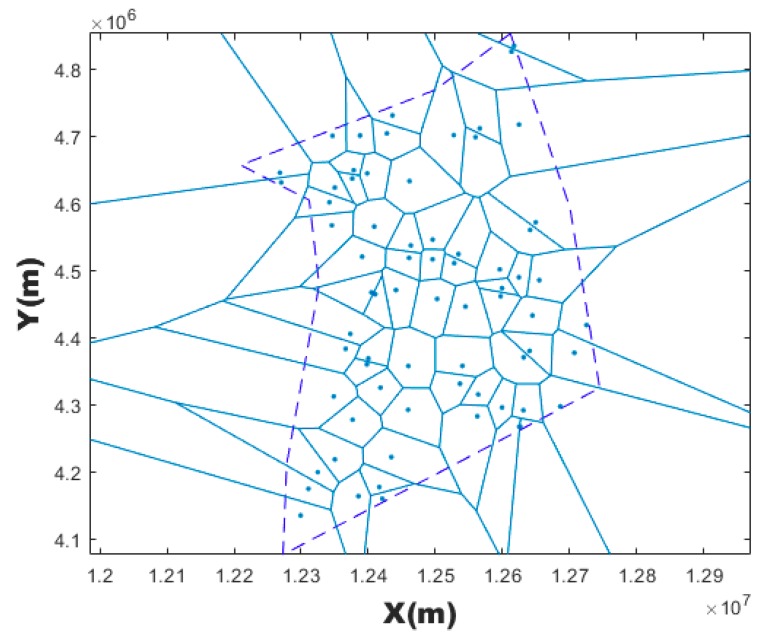
Random generating seeds.

Within the square airspace surrounding the airspace boundary, random point was generated. If this point falls within the airspace boundary, a random seed is successfully generated. Repeat this step over and over again to generate n generating points. Then the Voronoi diagrams can be generated. Figure 3 is an example of a Voronoi diagram generated by 90 randomly generating points.

#### 2.1.2. Seeds Were Taken Every $D_{min}$ Kilometers along Routes

**Figure 4:**
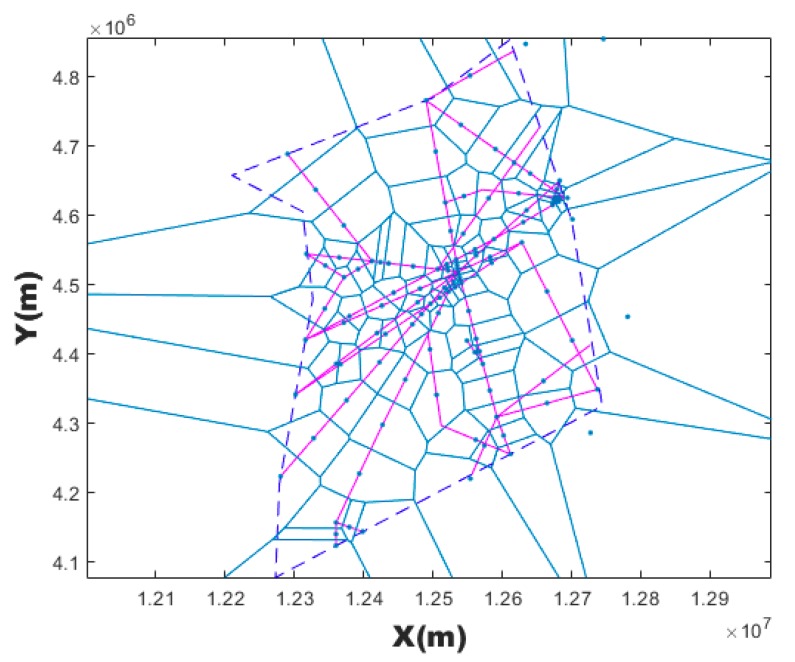
Seeds generated by taking a point every 30 km along the route.

Generating points for constructing VCs include starting and end points of every route, as well as some intermediate points along routes separated by at least $D_{min}$ kilometers. If the length of r segment is $D_{r}$, then the number of intermediate points equals the following:

(2) $$m_{r}=⎣\frac{D_{r}}{D_{min}}⎦-1,$$

where ⎣·⎦ is the operator of rounding down. Less $D_{min}$ means more VCs with small granularity when discretizing the airspace. Figure 4 is a Voronoi diagram generated by taking a point every 30 km along the route (pink coloring) as generating points.

#### 2.1.3. Hexagonal Seed

**Figure 5:**
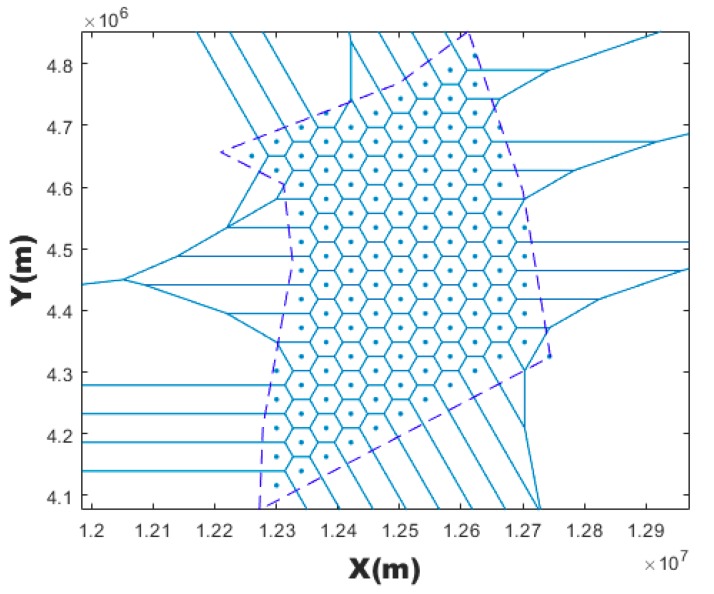
Hexagonal seed.

The generating points are evenly and staggeringly distributed in the studying airspace. The resulting Voronoi diagram is a regular hexagon. Figure 5 is an example of a hexagonal Voronoi diagram generated in the studying airspace.

### 2.3. Reconstruct Voronoi Cells According to the Airspace Boundary for Coloring Correctly

After discretizing the airspace with Voronoi cells (VCs), in order to visualize which sector these Voronoi cells belong to, the Voronoi diagram needs to be colored. Voronoi cell coloring in the airspace boundary can be easily colored by calling the fill or pitch function of MATLAB, but it is not easy to color Voronoi cells near the airspace boundary. Ordinary Voronoi diagrams have the following basic properties [56].

- All the points on the common edges have equal distances to their neighbor generating points.
- The generating point of VC is the closest generating point to any point within this VC.
- The line between two generating points is perpendicular to the common edge of the two Voronoi polygons.

These characteristics of VCs will be utilized to calculate the intersection point between the edge of the boundary and edge of the VC.

Reconstructing VCs and coloring all cells for visualization are very important for reconfiguration. We noticed that color could not be filled in some open VCs according to the index of which sector these cells belonged to. In the process of coloring, just the inner part of the VC crossing by airspace boundary is preferred to retain, so we developed a method to reshape these VCs in the next part for coloring all cells correctly.

The procedure for the solution of the intersection point between the edge of the boundary and the edge of the VC is defined as follows:

Firstly, all endpoints of the boundary line were sorted anticlockwise. The equation of each boundary line is derived from Table 1. The equation of a boundary line can be expressed as Equation (3). $k_{1}$ is the slope of this line and $b_{1}$ is the intercept of a straight line on y axis. According to the characteristic that all the points on the common edges have equal distances to their neighbor generating points, the common edges must be perpendicular to the line between two generating points, which generate VC that passed through by boundary segment. As a result, we can get the function of common edges as Equation (4). $k_{2}$ is the slope of this line and $b_{2}$ is the intercept of a straight line on y axis. The intersection point can be obtained from two of these linear equations.

(3) $$y=k_{1}x+b_{1}.$$

(4) $$y=k_{2}x+b_{2}.$$

**Figure 6:**
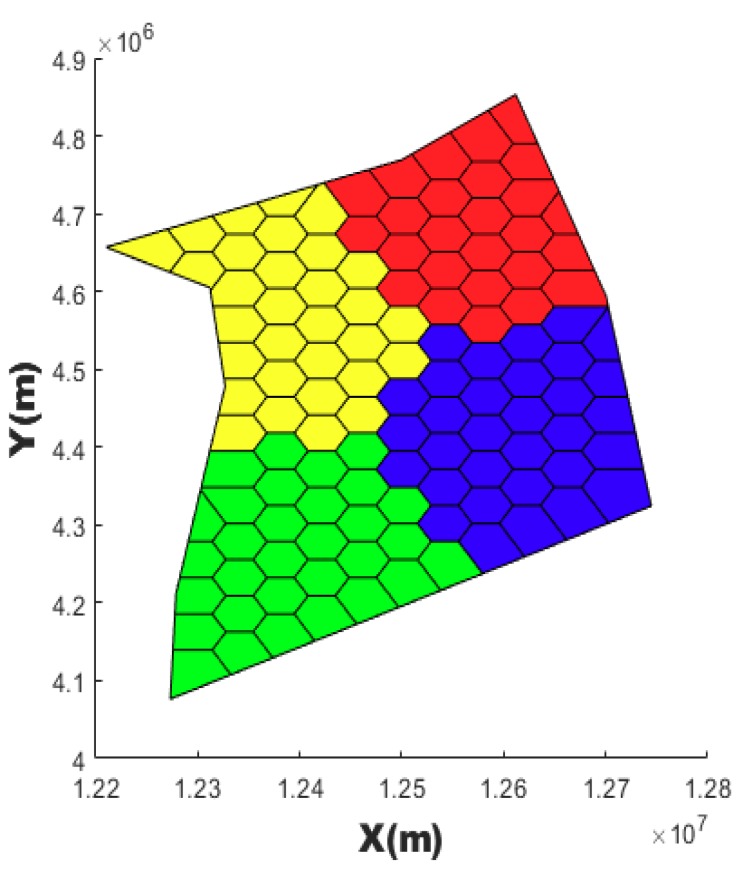
Voronoi cells are reconstructed and colored according to the airspace boundary.

Thereafter, only points within the airspace boundary of VC which is intersected by the boundary segment and intersection point are retained as the new vertices set of these VC. After anti-clockwise sorting of these new vertices sets of these VC, every VC can be correctly colored by the program. Figure 6 is an example that the Voronoi cell diagram is reconstructed and colored according to the airspace boundary. With this reconstruction and coloring, it is not only easy to distinguish which sector VCs belong to, but it is also easy to solve the intersection of the route and boundary VCs.

### 2.4. Task Load Measurement and Objective Function

The research focusing on the controller workload has been carried out by a desire to balance the task load, understand occupational stress, eliminate operational errors, enhance safety and improve throughput. Many workload calculation methods have been proposed in previous studies based on dependent variables: Physical activity [57], physiological indicators [58,59,60], simulation models of the controller’s tasks [46,47,61,62,63,64] and subjective ratings [65,66]. Gianazza [67] have reviewed literature about workload prediction and compared several machine learning methods on the problem of learning workload prediction models from historical data. Surprisingly, there is no globally accepted definition for the controller workload—“Controller workload is a confusing term and with a multitude of definitions, its measurement is not uniform” [68]. From the perspective of technical feasibility, the task load calculation method based on the statistical controller’s tasks in the field or simulated tests is easier than others.

Each sector is typically positioned with one or two controllers. A radar controller is responsible for radio communications with the aircraft, monitoring the radar screen to maintain safe separation and communicating with other controllers. When two controllers work a sector, the second is an associate controller, known as a data-controller. The data-controller typically receives the flight-plan information and helps plan and organize the flow of traffic within the sector. During exceptionally busy periods, a third controller may be assigned to the team, although three-member teams are not typically planned for. The task load is the sum of the time spent by controllers performing tasks [69].

Task load calculation model designed by Oktal and Yaman [64] considered three kinds of task load, the monitoring task load, the conflict task load and the coordination task load. The monitoring and the conflict task loads occur inside the sector while the coordination task load between the sector and its adjacent sector. Due to the fact that the air traffic controllers in China usually build an aerial overpass bridge at the intersection of the route to ensure that flights that may be conflicting have been transferred to the specified flight levels before they arrive at the intersection, it indicates that the monitoring task load includes the conflict task load. For this reason, our task load calculation model only considers monitoring and coordinating task loads. Formulas of our task load calculation model is similar to these designed by [47,49], while the tiny difference lies in that we consider the duration of the task, not just the number of flights in our task load model. We suppose that the monitoring task load of one aircraft is proportional to flight time. This ratio is the average time per unit of time that the aircraft needs to be monitored by the controller (including conflict resolution by telephone). The coordinated task load of an aircraft is equal to the average time for the controller to complete the takeover or handover when this aircraft cross sector boundaries.

#### 2.4.1. Task Load Measurement

Given that the airspace studied has *n* VCs, each $VC_{i},i\in \left\{1,\ldots ,n\right\}$, must be assigned to some sector $s_{j},j\in \left\{1,\ldots ,k\right\}$ where k is the number of desired sectors to be opened. If $VC_{i}$ is assigned to $s_{j}$ sector, then $x_{i}^{j}=1$, else 0. The relationship between task load of jth sector and task load of $VC_{i}$ is expressed as follow:

(5) $$wl^{j}=\sum_{i=1}^{n}x_{i}^{j}\cdot wl_{i},\forall j\in \left\{1,\ldots ,k\right\},\;\forall i\in \left\{1,\ldots ,n\right\}.$$

$wl_{i}$ includes two kinds of task loads, namely monitoring and coordinating task loads, as mentioned before.

(6) $$wl_{i}=wl_{i}^{mot}+wl_{i}^{cod}.$$

Monitoring the task load measurement:

Assume that there are m routes passing through $VC_{i}$. Then monitoring task load was calculated as follows:

(7) $$wl_{i}^{mot}=\alpha_{mot}\cdot \sum_{r=1}^{m}f_{r}\;\cdot (l_{i}^{r}/v_{r}).\;$$

$l_{i}^{r}$ is the length of route r in $VC_{i}$, km.

$v_{r}$ is the average speed of flights on route r, km/h.

$f_{r}$ is the hourly traffic volume passing through route r, number/h.

$\alpha_{mot}$ is the statistical monitoring time per hour per flight by field test, seconds/h.

**Figure 7:**
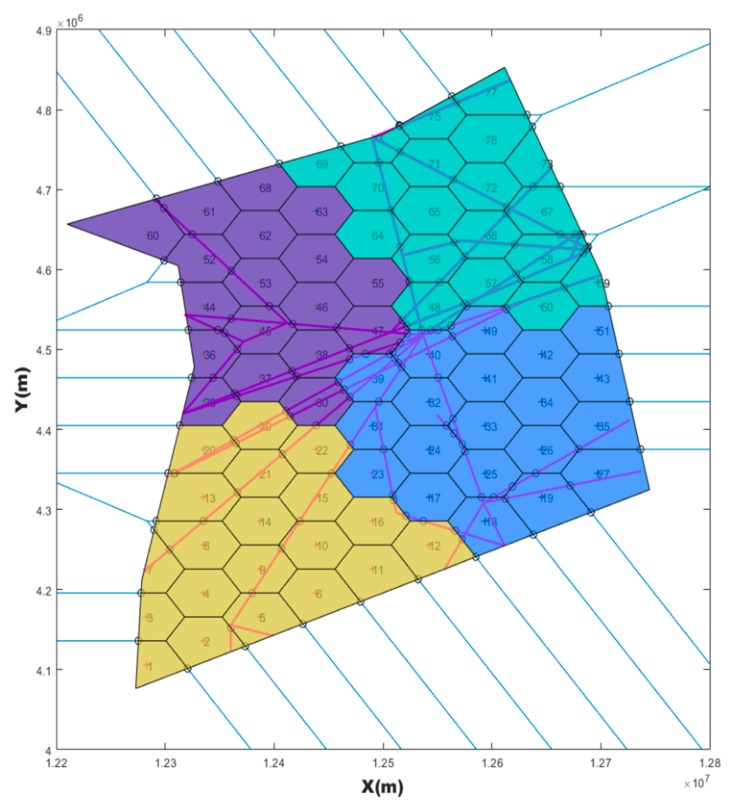
Intersection of all routes with the Voronoi cell (VC) they pass through.

The intersection of all routes with the VC they pass through is computed and stored in a structured array before the task load is computed, as shown in Figure 7.

The coordinating task load measurement:

The coordinating task load is calculated as follows:

If ∀r∈{1,…,m} passing through $VC_{i}$ and both of the boundaries of VC were the sector boundary, then:

(8) $$wl_{i}^{cod}=\alpha_{cod}\cdot \sum_{r=1}^{m}(\gamma_{r}^{i}\cdot f_{r}).$$

$f_{r}$ is the hourly traffic volume passing through route r, number/hour.

$\alpha_{cod}$ is the statistical coordinating time per hour per flight by field test, seconds/hour.

If ∀r∈{1,…,m} passing through $VC_{i}$ and two of the boundaries of $VC_{i}$ was the sector boundary, then $\gamma_{r}^{i}$ = 2. This rarely happens unless a single VORONOI diagram is large. If ∀r∈{1,…,m} passing through $VC_{i}$ and just one of the boundaries of $VC_{i}$ was the sector boundary, then $\gamma_{r}^{i}\;$= 1. If ∀r∈ passing through $VC_{i}$ and none of the boundary of VC was the sector boundary, then $\gamma_{r}^{i}\;$= 0.

#### 2.4.2. Objective Function

Four criteria were included in our objective function for the evaluation of a solution. The equation of objective function is:

(9) $$F=\;(\alpha_{1}\cdot cost_{imb}\;+\alpha_{2}\cdot wl_{tot}^{cod}\;+\alpha_{3}\cdot cost_{tot}^{SDT}+\alpha_{4}\cdot cost_{tot}^{rin}).$$

$\alpha_{1},\;\alpha_{2},\;\alpha_{3}\;\mathrm{and}\;\alpha_{4}$ represent the weight of different objective components.

Minimize the task load imbalance:

The first criterion minimizes the level of the task load imbalance among all controlled sectors in configuration. The task load imbalance of all controlled sectors is then computed with the following equation:

(10) $$cost_{imb}=\sum_{j=1}^{k}\left|wl_{j}-\mu \right|$$

where:

k: Number of desired sectors to be opened;

j: jth sector;

μ: Average task load, μ=$\frac{\sum_{j=1}^{k}wl_{j}}{k}$;

$wl_{j}$: Total task load in *j*th sector.

Minimize the total coordinating task load:

Coordinating task load is the key factor of the total task load. In the process of airspace sectorization, it is almost impossible to decrease the monitoring task load. A good airspace configuration plan must be a plan with the minimum coordination task load. Minimizing coordination task load can not only reduce the total task load, but also keep the sector consistent with the main traffic flow. The total coordinating task load, $wl_{tot}^{cod}$, is equal to the sum of task load of all VCs as following equation.

(11) $$wl_{tot}^{cod}\;=\sum_{i=1}^{n}wl_{i}^{cod},\;i\in \left\{1,\ldots ,n\right\}.$$

Minimize the cost of a short dwell time (SDT):

The sector capacity is calculated based on MAP (Monitor Alert Parameter) from the Federal Aviation Administration (FAA [70], which is roughly 5/3 of the average sector flight time (in minutes). This means that sector capacity increases with dwell time (average sector flight time). Since the airspace studied was an en-route airspace, we arbitrarily set the minimum of dwell time as 240 s. The cost of the short dwell time was measured as follows:

(12) $$cost_{tot}^{SDT}=\sum_{j=1}^{k}cost_{j}^{SDT}.$$

For any flight a∈{1,…,m} in sector *j*,

(13) $$cost_{j}^{SDT}=\sum_{a=1}^{m}\left\{\left[t_{dw}^{min}-t_{dw}\left(a,j\right)\right]\cdot \varepsilon_{dw}\right\}\;.$$

(14) $$t_{dw}\left(a,j\right)\;=t_{out}\left(a,j\right)-\;t_{in}\left(a,j\right).$$

$t_{dw}\left(a,j\right)\;$ is the ath flight’s dwell time $t_{dw}\left(a,j\right)$ in the *j*th sector and $t_{dw}^{min}$ was set as 240 s.

$\varepsilon_{dw}$ is the penalty coefficient of the short dwell time,

(15) $$\varepsilon_{dw}=\{\begin{array}{c} \sqrt[{6}]{exp\left[t_{dw}^{min}-t_{dw}\left(a,j\right)\right]},\;if\;\left[t_{dw}^{min}-t_{dw}\left(a,j\right)\right]>0\\ 0,\;\;\;\;\;\;\;\;\;\;\;\;\;if\;\left[t_{dw}^{min}-t_{dw}\left(a,j\right)\right]\leq 0 \end{array}.$$

Minimize the cost of reentering the same sector:

We hoped that every trajectory entered the same sector less than or equal to one time. To some extent, it means this cost will cause the optimization search process to discard those non-convex sectors and disconnected sectors.

(16) $$cost_{tot}^{rin}=\sum_{r=1}^{m}\;cost_{r}^{rin}.\;$$

(17) $$cost_{r}^{rin}=\sum_{j=1}^{k}\beta \cdot (t_{rin\_j}-1).$$

If r route does not pass through the j sector, then $t_{rin\_j}\;$ = 0; if r route passes through the jth sector more than once, the cost of r route reentering in the j sector will increase with reenter times. β is the reenter penalty factor. We arbitrarily set it as 100 s.

### 2.5. Constraints

Three constraints, task load, connectivity and compactness were considered.

#### 2.5.1. Task Load Constraint

In theory, the highest value for the R-side task load for a 15-min period is 900 s (15 min times 60 s/min), assuming (unrealistically) that a controller can effectively use all 900 s of available time and that a second controller is handling the D-side task load. When the task load rollup exceeds a certain threshold (around 600 s), it is assumed that two controllers are working the traffic [69]. The maximum acceptable task load in each sector is set as 3420 s for a 1-h period. Assuming that there is only one sector in the studying airspace at first, the task load increases gradually with time. Assuming that there is only one sector in the research airspace at first, the task load will gradually increase with time. When the task load increases to its maximum, the partition of the airspace is triggered. In order to balance the workload, the best partitioning result is that the workload of each sector is half of the maximum workload. Over time, when the task load of both sectors reaches the maximum task load, the best division is that the task load of each sector is 2/3 of the maximum task load. It can be inferred that when airspace is divided into *k* sectors, the optimal average task load can be expressed as the following:

(18) $$\mu =\frac{k-1}{k}\cdot wl_{max}$$

When the airspace keeps *k* sectors unchanged, and the traffic flow of different sectors is not consistent with the growth of time, the sector structure also needs to be adjusted. Before changing the number of sectors from *k* to *k* + 1, the variation range of the average task load is as follows:

(19) $$\frac{k-1}{k}\cdot wl_{max}\leq \mu \leq wl_{max}$$

Equations (18) and (19) are based on the trigger mechanism of sector division, which is when the traffic flow of all sectors increases to $wl_{max}$. The real environment may be that the traffic flow growth in each sector is not balanced, some sectors may not grow, and some sectors have reached the maximum. It also needs to be adjusted at this time. The more sectors there are, the more likely the growth imbalance will be. Then the trigger mechanism will fail. In the case of an unbalanced growth of traffic flow in each sector, it is necessary to adjust the sector structure as long as a single sector reaches its maximum. In this case, the task load of the busiest sector can usually be reduced by adjusting the structure of airspace without increasing the number of departments. Assuming this happens, there are currently k sectors, the total workload is $wl_{tot}$ and the average workload is $wl_{tot}/k$. Then, the following equation is used to constrain the task load of each sector.

(20) $$\beta_{1}\cdot wl_{tot}/k\leq wl_{i}\leq \beta_{2}\cdot wl_{max}.$$

$\beta_{1}$ is the minimum deviation from the average of total task load. The smaller the value of $\beta_{1}$ is, the more feasible solutions are.$\;\beta_{2}$ is the design redundancy parameter. If the design cycle is longer, the $\beta_{2}$ should be smaller, leaving enough room for traffic flow growth.

#### 2.5.2. Connectivity Constraint

The connectivity constraint ensures that the sectors are not fragmented [28]. In other words, a sector must be a contiguous portion of airspace. Connectivity of a sector was assured by checking whether every VC in this sector has at least one common edge with another VC or not. If each VC in a certain sector has no common edge with another VC, then this sector with this VC is not connective.

Definition: Undirected graph D=(VC, E), VC set VC = {$VC_{1}$*,* $VC_{2}$*,...,* $VC_{n}$} belongs to sector $s_{i}$, if two Voronoi cell have a common edge between each other, then the two cells are adjacent. Define the adjacency matrix M = ${\left(M_{ij}\right)}_{n\times n}$ as follows:

$$M_{ij}=\{\begin{array}{c} 0\;,if\;VC_{i}\;to\;VC_{j}\;\mathrm{is}\;\mathrm{not}\;\mathrm{adjacent}\\ 1\;,if\;VC_{i}\;to\;VC_{j}\;\mathrm{is}\;\mathrm{adjacent}\; \end{array}$$

Similarly, we define the reachable matrix R  = ${\left(R_{ij}\right)}_{n\times n}$ of graph D as follows:

$$R_{ij}=\{\begin{array}{c} 0\;,if\;VC_{i}\;to\;VC_{j}\;\mathrm{is}\;\mathrm{not}\;\mathrm{reachable}\\ 1\;,\;if\;VC_{i}\;to\;VC_{j}\;\mathrm{is}\;\mathrm{reachable}\; \end{array}$$

R can be calculated by the adjacency matrix M [71,72].

If the sum of every column of R equals *n*, then all VCs in sector $s_{i}$ are connected, otherwise, the conclusion is contrary.

Two methods are used to guarantee connectivity. One is to reject the new solution directly, keep the original solution and search on the basis of it. The other is to repair the disconnected sector into connected sector through the following methods. According to the adjacency matrix of a sector, the numbers of connected areas were calculated in this sector. If there are more than two connected areas in any sector, only the area with the largest number of VCs are reserved, and the other isolated area is allocated to some sector with the smallest task load and this sector must be adjacent to these isolated areas. When the simulated annealing temperature is higher, connectivity of sectors will be repaired with a larger probability. On the contrary, when the temperature is low, connectivity of sectors will be repaired with a small probability. While these disconnected solutions were rejected with a larger probability.

#### 2.5.3. Compactness Constraint

In the process of using the neighborhood search method to generate new solutions, there are a few VCs very far from their assigned sector. This is called non-compactness. Compactness of a sector is defined as follows.

(21) $$\delta_{j}=\sum_{i=1}^{n}\frac{D_{i}^{j}\cdot z_{i}^{j}}{n}\forall i\in \left\{1,\ldots ,n\right\},\forall j\in \left\{1,\ldots ,k\right\}.$$

$D_{i}^{j}$ is the distance between generating point of $VC_{i}$, and the center of $s_{j}$. If $VC_{i}$ located in sector $s_{j}$, $\;z_{i}^{j}=1$, otherwise, $\;z_{i}^{j}=0$. $\delta_{j}$ is the average distance of all VC to the center of $s_{j}$. The average distance is set as the threshold to assure the compactness of sectors.

Firstly, all VCs that distance between $\mu_{j}$ and themselves were larger than the threshold value were found. Then, the numbers of connected regions were found out in the undirected graph composing of these cells. Each of them was allocated unconditionally to the nearest sector.

#### 2.5.4. Number Constraint of Flexible Vertices

**Figure 8:**
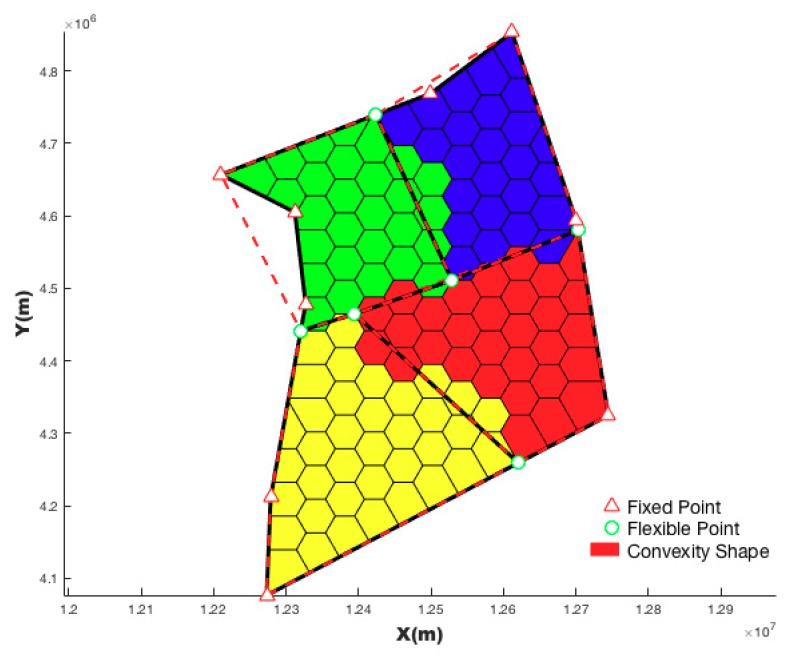
Flexible and fixed vertices.

The vertices of a sector boundary can be classified according to their sharing by multiple sectors. All vertices of a sector boundary are divided into two categories: Flexible vertices and fixed vertices. The classification of vertices is illustrated in Figure 8.

The blue-coated sector has a vertex shared (two-zone shared vertex) with the red-coated sector, as well as with the green-coated sector. There is a vertex shared by the blue-coated sector, the red-coated sector and the green-coated sector. Both of the two-zone shared vertex and three-zone shared vertex are called flexible vertices. The other vertices marked with a red triangle in the blue-coated sector in Figure 8 were fixed. We found that when the number of flexible vertices (NFV) in a sector was less than three, the sector shape generated by SAA would inevitably appear C shaped. Therefore, we had to force the NFV to be greater than three. This constraint was not used for the SAA search process, but only for the sector evaluation and post-processing. If the number of flexible points in the sector generated by SAA was less than three, the SAA process was repeated.

## 3. Solution Method

**Figure 9:**
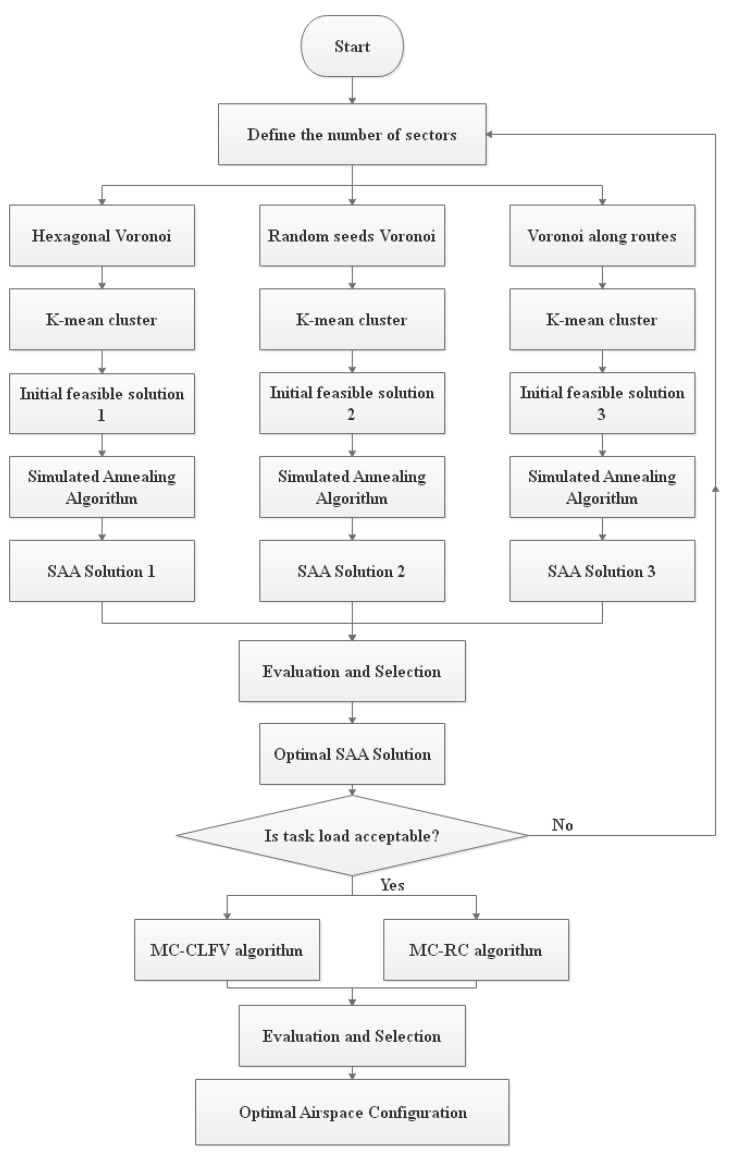
Framework of the solution method.

There are three common design questions related to meta-heuristics: The representation of solutions handled by the algorithm, new solution generation method and the definition of the objective function that will guide the search. The definition of the objective function has been discussed in the previous section. In this section, we will introduce the representations of solutions and solution method of airspace reconstruction problem. The framework of the solution method is shown in Figure 9.

### 3.1. Solutions Expressions

The representation was based on the proposed reconfiguration problem modeling. When calculating the task load, the representation of solution was similar to the assignment problem. If $VC_{i}$, ∀i∈{1,…,n}, is assigned to sector $s_{j},j\in \left\{1,\ldots ,k\right\}$, then $z_{i}^{j}=1$, otherwise $z_{i}^{j}=0$. Here, an example is given to show the representation of this solution. Suppose there are 10 cells allocated to three sectors.

The first type of representation of solution $z=\left\{\begin{array}{c} z_{1}^{1},z_{2}^{1},\ldots z_{n}^{1}\\ z_{1}^{2},z_{2}^{2},\ldots z_{n}^{2}\\ z_{1}^{3},z_{2}^{3},\ldots z_{n}^{3} \end{array}\right\}$ was shown in Table 2. If VCs (1, 2, 3) are adjacent, as well (4, 5, 6) and (7, 8, 9, 10), then the solution show in Table 2 is a feasible one. This type of representation of the solution was used to calculate the task load.

The second type of representation of solution $x=\{x_{1},x_{2},\ldots ,x_{n}\}$ was shown in Table 3. This type of representation of the solution was used to renew the current solution.

Two kinds of these representations can be transformed into each other.

### 3.2. K-Means Clustering to Generate the Initial Feasible Solution

Given a set of observations ($p_{i}$ is coordinate of generation point of ${\mathrm{VC}}_{i}$, i = 1, 2, ..., *n*), *k*-means clustering [73] aims to partition the n observations into *k* (≤n) sets s = {$s_{1}$*,* $s_{2}$*, …,* $s_{k}$} so as to minimize the within-cluster sum of squares (i.e., variance). Formally, the objective is to find:

(22) $$\begin{array}{c} \arg min\;\\ S \end{array}\;\sum_{j=1}^{k}\sum_{p_{i}\in s_{j}}{\left|\left|p_{i}-\mu_{j}\right|\right|}^{2},$$

where $\mu_{j}$ is the centroid of points in $s_{j}$.

After *k*-means clustering, the sector of ${\mathrm{VC}}_{i}$ will become clear. The second type of representation of the solution was used to represent *k*-means clustering results. For example, x = [1, 1, 1, 2, 2, 3, 3], means ${\mathrm{VC}}_{1}$, ${\;\mathrm{VC}}_{2}{\;\mathrm{and}\;\mathrm{VC}}_{3}$ belong to sector 1, ${\mathrm{VC}}_{4}{\;\mathrm{and}\;\mathrm{VC}}_{5}$ belong to sector 2, ${\mathrm{and}\;\mathrm{VC}}_{6}\;{\mathrm{and}\;\mathrm{VC}}_{7}\;$ belong to sector 3.

As mentioned in Section 2.2 above, the solution space of different discretization methods (even the number of VCs) in the studied airspace were different. Therefore, we designed three parallel approaches to explore the optimal solution. Finally, the best solution was chosen by comparison. This process is shown in Figure 9.

### 3.3. Neighborhood Search Strategy

#### 3.3.1. Variable Neighborhood Search

To prevent local optimization, the following two variable neighborhood search methods were used in the search process.

**Figure 10:**
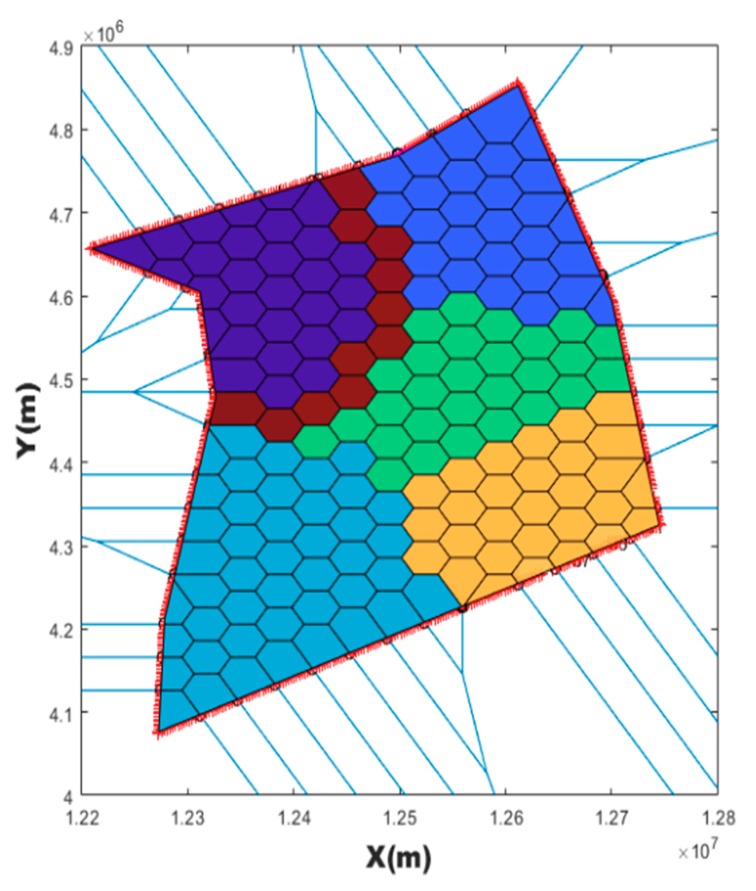
An example of the propagable neighborhood (crimson) of the sector (dark blue) with the smallest task load.

Algorithm 1, RC_PNB search algorithm:

Firstly, we needed to calculate the workload of all sectors and find out the sectors with the minimum task load. Assuming that the sector with the minimum task load was the sector $s_{j}$, and $\;VC_{i1}$*,*$\;VC_{i2}$*,*$\;VC_{i3}$*…*$VC_{iA}$ (all Voronoi cells with crimson color of dark blue sector with the smallest task load as showed in Figure 10) adjacent to at least one of the VC in the sector $s_{j}$, we defined set {$VC_{i1}$*,*$\;VC_{i2}$*,*$\;VC_{i3}$*…*$VC_{iA}$} as $PNB_{s_{j}}$, propagable neighborhood of $s_{j}$. These VC of $PNB_{s_{j}}$ might be distributed in one sector or multiple sectors.

A variable neighborhood search algorithm was used according to a certain probability as following.

**Algorithm 1** RC_PNB Search Algorithm
x
*=*
$\;x_{0}$
$PNB_{s_{j}}=PBN\_find\_fun\left(x,M,sn_{tl\_min}\right)$;$A=length\left(PNB_{s_{j}}\right)$;B=randi(A);
*If rand > 0.5*
  $A_{1}$*=* A(1:B,:);
*else*
  $A_{1}$*=* A(B:end,:);
*end*
           $x\left(A_{1}\right)=sn_{tl\_min}$

*Connectivity constraint check and assurance algorithm,*
 x
*→*
$x_{1}$

*Compactness constraint assurance,*
$\;x_{1}$
*→*
$x_{2}$

*If*
$F\left(x_{2}\right)$
*<*
$\;F\left(x_{0}\right)$
  
$x_{0}$
*=*
$\;x_{2}$

*else*
  
$x_{0}$
*=*
$x_{0}$

*end*


Algorithm 2, PN_VC search algorithm:

All VCs adjacent to other sectors in the sector $s_{j}$ with the lowest task load are called expandable boundaries points (EBP). A neighborhood search algorithm of expandable boundary points (EBP_NB search algorithm) in minimum task load sector $s_{j}$ was developed as following.

**Figure 11:**
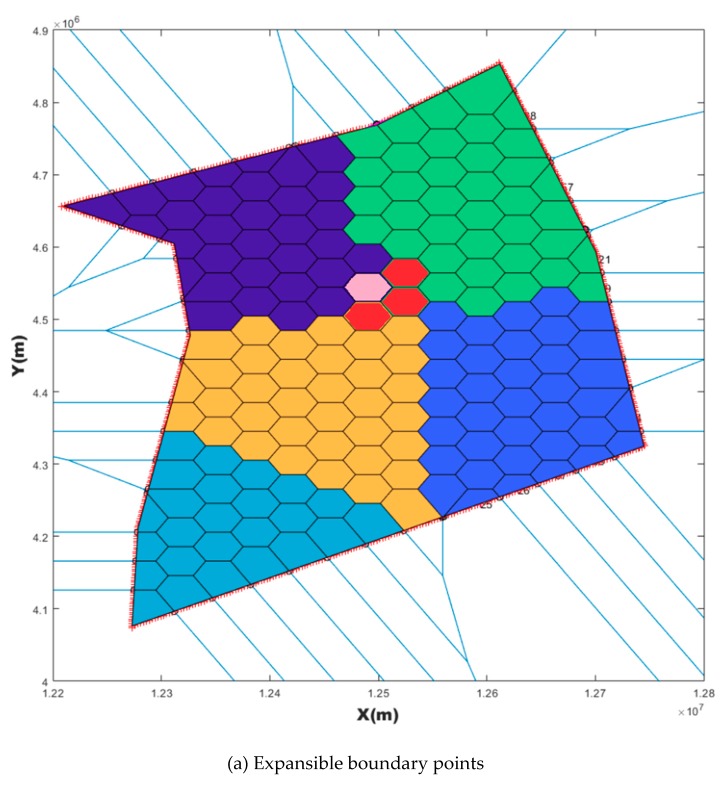

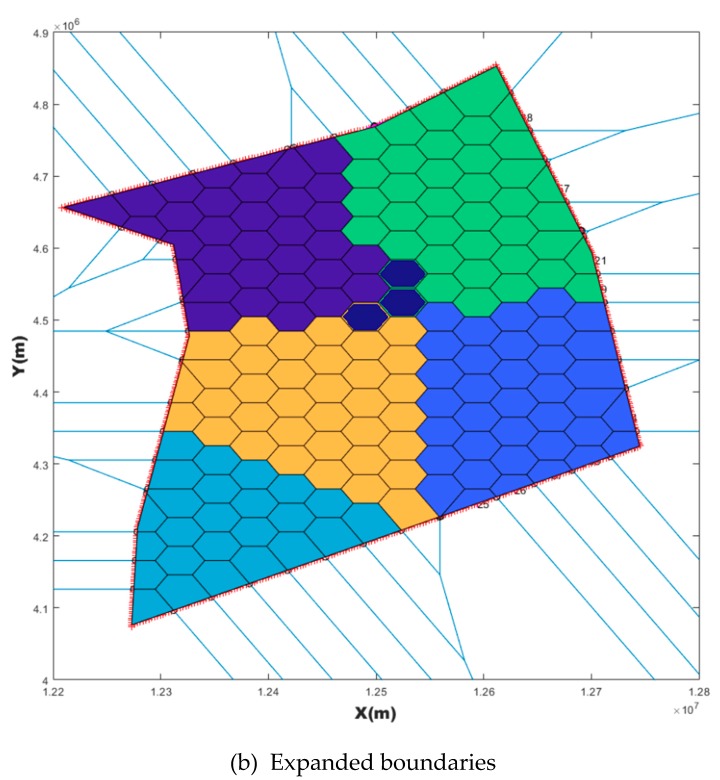
An example of expansible boundary points (**a**) and expanded boundaries (**b**) in PN_VC search.

As in Figure 11a, one VC (pink color) in EBP was randomly selected, all VCs around this VC but not belonging to $s_{j}$ (red color) were called expandable boundary points of this VC (EBP_VC). A neighborhood search algorithm of propagable neighborhood of VC (PN_VC search algorithm) in minimum task load sector $s_{j}$ was developed as following.

**Algorithm 2** PN_VC Search Algorithm
x
*=*
$\;x_{0}$

*Random select a VC in EBP_VC*

*Check out PN_VC of this VC*

*Allocate PN_VC to*
$s_{j}$
*and update*
x

*Connectivity constraint check and assurance algorithm,*
 x
*→*
$x_{1}$

*Compactness constraint assurance,*
$\;x_{1}$
*→*
$x_{2}$

*If*
$F\left(x_{2}\right)$
*<*
$\;F\left(x_{0}\right)$
 $x_{0}$
*=*
$\;x_{2}$

*else*
 $x_{0}$
*=*
$x_{0}$


#### 3.3.2. Connectivity Constraint Check and Assurance

After calculating the reachable matrix *R* of the sector, if the sum of each column of *R* is less than *n*, all connected components of the sector should be detected. If a sector is divided into several blocks by using the neighborhood search method, only the largest blocks in the sector are retained, other smaller blocks are unconditionally allocated to the sector connected to these components with the smallest distance.

#### 3.3.3. Compactness Constraint Assurance

In order to ensure the compactness of the sector, the average distance from the generating points of VCs to the center point of all VCs was set as a threshold to assure compactness of sectors. If some VCs were far from their assigned sector more than a threshold in Equation (21), they would be allocated to the adjacent sector with the smallest distance; otherwise, they would be kept in the original sector.

### 3.4. Simulated Annealing Algorithm

Simulated annealing starts from a state $x_{0}$ and continues until temperature T cools down to $\;T_{min}$ at a cooling rate σ. In the process, the call neighbor (x) should generate a randomly chosen neighbor of a given state x; to increase the search space, repeat call neighbor (x) *L* times at a specific temperature. The call random (0, 1) should pick and return a value in the range [0, 1], uniformly at random. The algorithm determines whether the new solution $x_{new}$ is better or worse than the current solution x. If the new solution is better than the current solution, it becomes the next solution. If the new solution is worse than the current solution, the algorithm can still make it the next solution. The algorithm accepts a worse solution based on an acceptance function 1/(1 + $e^{\frac{\delta }{T}})$. Since both δ = *E*($x_{new})$ − *E*(x) and T are positive, the probability of acceptance is between 0 and 1. Lower temperature leads to smaller acceptance probability. At the same time, larger δ leads to smaller acceptance probability. The best solution and value of objective function were recorded at every specific temperature. After the simulated annealing, the corresponding solution of the minimum value of all objective function at different temperatures was extracted as the optimal solution. The following pseudo code presents the details of the simulated annealing algorithm:

**Algorithm 3** Simulated Annealing Algorithm*Let*x*=*$x_{0}$. *n = length(*$x_{0}$)
$T=T_{0}$

*M = 100, % m is the iteration times at different temperatures*
$T_{min}$ = 1e-3
*Partition = [[];*

*While*
T
*>*
$\;T_{min}$

*Temp = zeros(m,n + 1);*

*for k = 1:m*

*If rand(1) > 0.6*
*RC_PNB search algorithm,*$x_{new}$*←neighbor(*x)
*else*
*PN_VC search algorithm,*$x_{new}$*←neighbor(*x)
*end*
*If F(*$x_{new}$*) < F(*$x_{0}$)
x
*←*
$x_{new}$

*Elseif /(1+*
$\;e^{\frac{F\left(x_{new}\right)-\;F\left(x_{0}\right)}{T}}$
*) ≥ random(0, 1):*

x
*←*
$x_{new}$

*End*
*temp(k,:) = [* x*, F(*x)]; 
*end*
*[*$F_{min}$*,index] = min(temp(:,end)); %Find the optimal partition at current temperature*.
*Partition = [partition; temp(index,end)];*

T
*=*
 T×σ; here σ
*is cooling rate*

*End*

*[*
$F_{opt}$
*,index1] = min(Partition (:,end));*

$x_{opt}$
*= Partition (index1,1:end-1); %the final state is*
$x_{opt}$
*, optimal value of objective function is*
$F_{opt}$


### 3.5. Post-Processing

After solving the model by SAA, the boundaries of each sector are obtained. Even considering the compactness of the objective function, sector boundaries generated by SAA, like all bottom-up methods, still appear as zigzag, ladder or C-shaped. Drew [41] used the Douglas-Peucker (DP) algorithm to smooth shared boundaries of the sectors generated by mixed integer programming, there are still some obvious problem, such as boundaries with a large zigzag shape. Li, Wang, Hwang and Hwang [29] also developed a bisection method based on the shortest path to smooth boundaries of the sectors. How these methods ensure the balance of task load among sectors is questionable, because they adjust boundaries based on distance parameters rather than task load. However, the most critical criterion for sector division is task load balancing, because only task load balancing can give each sector the opportunity (buffer) to increase traffic throughput.

Generally, a convex hull can be constructed for each pair sectors generated by SAA (or any other methods). Then, there must be a line that can bisect this convex hull into two sectors with the balance task work. We found that when all these lines are drawn, these lines cannot clearly divide the studied airspace into the required number and shape of sectors. Therefore, this method was abandoned.

To illustrate the principle of our algorithm, we first observed the boundary characteristics of blue-coated sectors from Figure 8. Taking the blue-coated sector shown in Figure 8 as an example, we called the boundary of the studied airspace as the outer boundary, and the irregular boundary between the blue-coated sector and the adjacent (red or pink) sector as the shared boundary. Our algorithm was to smooth these irregular shared boundaries, and try to ensure convexity and task load balance of all sectors, as well as connectivity and compressibility. Next, all fixed and flexible vertices (described in Section 2.5.4) in each sector are found out by repeating this process. If all boundary vertices are connected by a straight line, the sector that coincides with the original sector can be obtained. However, in this way, the task load of the sector will be different from that of the original sector, resulting in unbalanced task load of the sector. Therefore, our idea was to reposition these flexible vertices and rebuild sector boundaries to balance workload sectors.

In order to guarantee the near-convexity of sector shape in the process of rebuilding sector boundaries, we added a near-convexity evaluation function ($cost_{tot}^{cvx})$.

(23) $$cost_{tot}^{cvx}=\;\sum_{i=1}^{k}C_{i}^{cvx}.$$

(24) $$C_{i}^{cvx}=\frac{SA_{i}^{cvx}-SA_{i}}{SA_{i}^{cvx}}.$$

$SA_{i}^{cvx}$ is the area of the convex hull of the vertex of the ith sector, in square meters.

$SA_{i}$ is the area of the ith sector, in square meters.

$C_{i}^{cvx}$ is the concave coefficient of shape for the ith sector. The smaller $C_{i}^{cvx}$ is, the less concave the polygon is and the closer it is to the convex shape.

$cost_{tot}^{cvx}$ is the concave coefficient of shape for all sectors. The smaller $cost_{tot}^{cvx}$ is, the better the overall convexity of all sectors is. In this way, this performance parameter can be added to Equation (9) to form the following new evaluation function, which can be used to evaluate the new sector generated by the random change of the position of these flexible vertices.

(25) $$F=\;\alpha_{1}\cdot cost_{imb}\;+\alpha_{2}\cdot wl_{tot}^{cod}\;+\alpha_{3}\cdot cost_{tot}^{SDT}+\alpha_{4}\cdot cost_{tot}^{rin}+\;\alpha_{5}\cdot cost_{tot}^{cvx}.$$

To prevent a concave shape, we added a constraint that the angle between all adjacent lines (counterclockwise) connected to the three-share point should not be greater than 180 degrees. In the MC-CLFV algorithm, this kind of constraint is called concave shape checking.

In order to prevent sector boundaries from getting too close to some important points (such as large traffic flow routes, intersections, airports, etc.), the minimum distance constraint between departmental boundaries and important points is added [29]. In the MC-CLFV algorithm, this kind of constraint is called minimum distance checking.

#### 3.5.1. MC-CLFV Algorithm

As mentioned earlier, the key to smooth the boundary is to locate these flexible vertices. Therefore, the problem of sector boundary smoothing becomes the problem of locating these flexible vertices. Monte Carlo techniques can be used to ensure high quality, robust designs [74,75]. In order to improve the search efficiency, we developed a dynamic Monte Carlo method by changing the location of flexible vertices (MC-CLFV).

Given all flexible vertices ($VF_{1}^{s_{i}},VF_{2}^{s_{i}},\ldots ,VF_{n1}^{s_{i}}$) belong to set VF, and all fixed vertices ($VD_{1}^{s_{i}},\ldots VD_{n2}^{s_{i}})$ belong to set *VD*. B is boundary of airspace.  L is the air routes passing by airspace. Then the shape of airspace, *G*, is a function of the above vectors, which can be defined as follows.

(26) G= g(VF,VD,B,L)

f is the vector of flow on air routes L. Then the objective function (25) under the specific sector shape is the function of traffic flow and shape, which can be expressed as the following.

(27) F= F(G, f)

Before describing the algorithm, we give the following definitions.

Γ is the standard deviation of task load for all sectors.

$\Gamma_{0}$ is the initial the standard deviation.

$F^{0}$ is the value of evaluation Equation (27).

$r_{0}$ is base search radius, which is equal to half of the average distance between all flexible vertices.

In search process of the MC-CLFV algorithm (Algorithm 4), a random location point around each flexible vertex within the radius is used to replace the location of these flexible vertexes in VF. Then *G* and *F* change with locations of these flexible vertices.

The search process of the Algorithm 4 is as following.

**Algorithm 4** MC-CLFV Algorithm
*Check out all flexible vertices*
$VF_{1}^{s_{i}},VF_{2}^{s_{i}},\;\;\ldots ,VF_{n1}^{s_{i}}$
*and all fixed vertices*
$VD_{1}^{s_{i}},\ldots VD_{n2}^{s_{i}}$
*in*
$s_{i},i=1,\;\;\ldots ,\;k$
*, sorted them counter-clockwise*
*Setting base search radius*, $r_{0}$, *and its decrease rate in every iteration,*$\sigma^{1}$*= 0.96*
*Calculate the value of the evaluation function,*
$\;F^{0}$
*;Calculate initial the standard deviation*
$\Gamma_{0}$
*While*$\Gamma_{0}>\tau$ (τ
*is maximum acceptable standard deviation*)  $r=r_{0}\;\times \sigma^{1}$
  For i = 1:length(VF)    θ = 2π× rand()    x($VF_{i}^{s_{i}}\;$) = x($VF_{i}^{s_{i}}$) +  r × rand() × cos θ;    y($VF_{i}^{s_{i}}\;$) =y($VF_{i}^{s_{i}}$) +  r × rand() × sin θ;  end  ***VD***
*and renewed*
VF*, constitute a new shape of sector.*
  *If concave shape checking or minimum distance checking is not true*    *Continue;*  *end*  ***G***
*= g*(VF,VD,***B***,***L***)  *Calculate F* = *F*(G***,*** f)  ΔF
*=*
$F^{0}-F_{opt}$
  $\Gamma =\sqrt{\frac{1}{k}\overset{k}{\underset{j=1}{\sum }}{\left(F_{j}-\bar{F}\right)}^{2}}$
  *If*
ΔF≤0
*and*
$\Gamma \leq \Gamma_{0}$
*and*
$\beta_{1}$
*× mean*($F_{j},j=1\ldots k)\leq$. *min*($F_{j},j=1\ldots k$. ) *and max*($F_{j},j=$. 1…k) ≤
$\beta_{2}$
*×*
$wl_{max}$
    $F_{opt}$
*=*
$F^{0}$
    $\Gamma_{0}=\;\Gamma$
    $G_{opt}$ = **G**  *end*
*end*


#### 3.5.2. MC-RC Algorithm

When some sector produced by SAA is surrounded by other sectors (hub-sector), the MC-CLFV algorithm cannot partition airspace properly. Especially, when traffic flow is crossed in the center of the airspace, or when an important airport is located in the airspace center. Basu, Mitchell and Sabhnani [43] also developed a ‘Pie Cutting’ method for more flexibility during sectorization. However, they did not specify what airspace the pie-cut method would apply to. Traffic flows and routes in different airspace have different characteristics, so the shape or partition method of the sector should be different. The concept of generic sectors proposed by the Federal Aviation Administration (FAA) supports this view. Generic sectors are defined as segments of airspace in air route traffic control centers (ARTCCs) that controllers could manage without significant specialized training or experience, beyond what they would normally acquire to become certified [76,77]. The following Monte Carlo method by radius changing (MC-RC) was developed for this type of situation.

The sector whose geometric center is closest to the geometric center of the airspace is chosen as the hub-sector. A circle, which its center is the geometric center of hub-sector and radius is the average distance between center and points consisting of the convex hull of the hub-sector, is constructed. By adjusting the radius, the task load of the circular region can be equal to that of the original sector.

**Figure 12:**
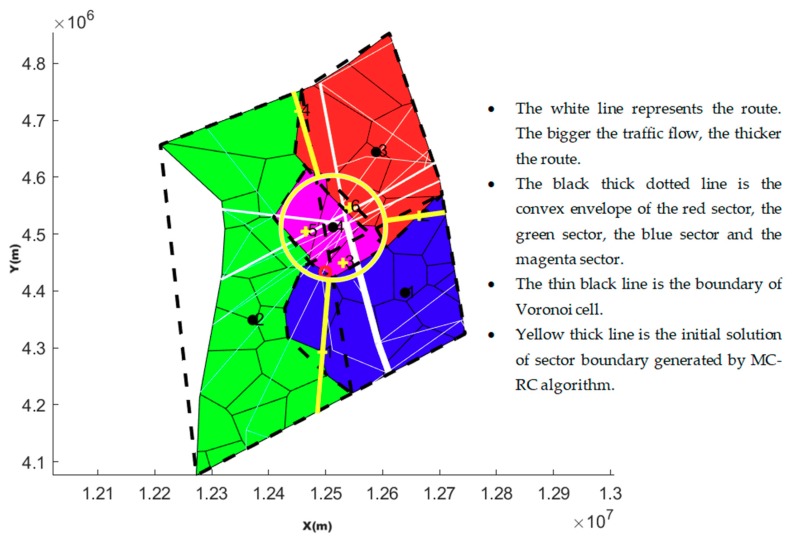
Initializing hub and spoke.

There is an overlapping region between two convex hulls of adjacent sectors. A line, which passes through the center of the hub-sector and the center of the overlapping region, is called the spoke. The spoke will divide the overlapping areas into two adjacent sectors. In theory, near the overlapping area of the convex hull, there must be a spoke that can divide task load of two sectors equally. Such a spoke can be found by keeping the starting point of the spoke (center of the hub-sector) unchanged and only changing the bearing of spoke from center. Figure 12 shows the initializing hub and spoke.

The search process of the MC-RC algorithm (Algorithm 5) is as following.

Configuration G is determined by dynamic variables $r_{h}$, $\theta =\left(\theta_{1},\theta_{2},\;\;\ldots ,\theta_{k-1}\right)$ and static variables B, L. k is the number of sectors. Graph can be expressed as $G=g\left(r_{h},\theta ,B,L\right)$.$\;r_{h}$ is the radius of the hub sector.  θ is the vector consisting of a three bearing of the spoke.  B is the boundary of airspace.  L are air routes passing by airspace. Then the value of the objective function in Equation (9) can be expressed as F=F(G,f).  f is the vector of flow on air routes L. Given that the value of the objective function of every sector is $F_{j},$ j=1, 2,…, k, under configuration G and traffic flow  f. Then $F^{ave}$ is the average of value of task load of all sectors. Given that $\;F^{h}$ is the value of the task load of the hub sector, then the task load difference between $F^{h}$ and $F^{ave}$ can be expressed as the following.

(28) $$\delta_{h}=F^{ave}-F^{h}.$$

If $\delta_{h}>0$, $\;r_{h}$ will increase by $\frac{r_{h}\cdot \delta_{h}}{F^{ave}}$ to make $F^{h}$ equal to $F^{ave}$, and vice versa. Therefore, $\;r_{h}^{i}$ at ith iteration can be expressed as the following.

(29) $$r_{h}^{i}=r_{h}^{\left(i-1\right)}\left(1+\frac{\delta_{h}}{F^{ave}}\right).$$

Γ is used to represent the standard deviation of the objective function. The pseudo-codes for solving $r_{h}^{i}$ and $\theta^{i}$ by the Algorithm 5 is shown as the following.

**Algorithm 5** MC-RC Algorithm
*Initializing*
$r_{h}^{0}$
*,*
$\;\theta^{0}$
*,*
$\;\delta_{h}$
*,i = 0*

*Calculating configuration parameters,*
$\;G^{0}=g\left(r_{h}^{0},\;\theta^{0}\;,B,L\right)$

*Calculating of value of objective function,*
$\;F^{0}=F\left(G,f\right)$

*While*
$\delta_{h}$
*≥ 50 or*
Γ
*≥*
 τ
   $r_{h}^{i}$
*=*
$r_{h}^{\left(i-1\right)}\left(1+\frac{\delta_{h}}{F^{ave}}\right)$
   *Generate three random integers between 0 and 360 and assign them to*
$\theta^{i}$*, n mean at*
i th
*iteration.*   $G^{i}=g\left(r_{h}^{i},\theta^{i},B,L\right)$.   *If minimum distance checking is not true*     *Continue;*   *end*   $F^{i}=F\left(G^{i},f\right)$
   $\delta_{h}$
*=*
$F^{ave}-F^{h}$
   $\Gamma =\sqrt{\frac{1}{k}\overset{k}{\underset{j=1}{\sum }}{\left(F_{j}-\bar{F}\right)}^{2}}$
   i
*= i + 1;*  *If*
$F^{i}$
*<*
$F^{0}$ and $\delta_{h}$ < 50 and Γ
*<*
 τ
*and*
$\beta_{1}$
*× mean*($F_{j},j=1\ldots k)\leq$*min*($F_{j},j=1\ldots k)$
*and max*($F_{j},j=1\ldots k$) ≤
$\beta_{2}$
*×*
$wl_{max}$
      $F^{0}$
*=*
$F^{i}$
      $G^{opt}$
*=*
$G^{i}$
   *end*
*End*


**Figure 13:**
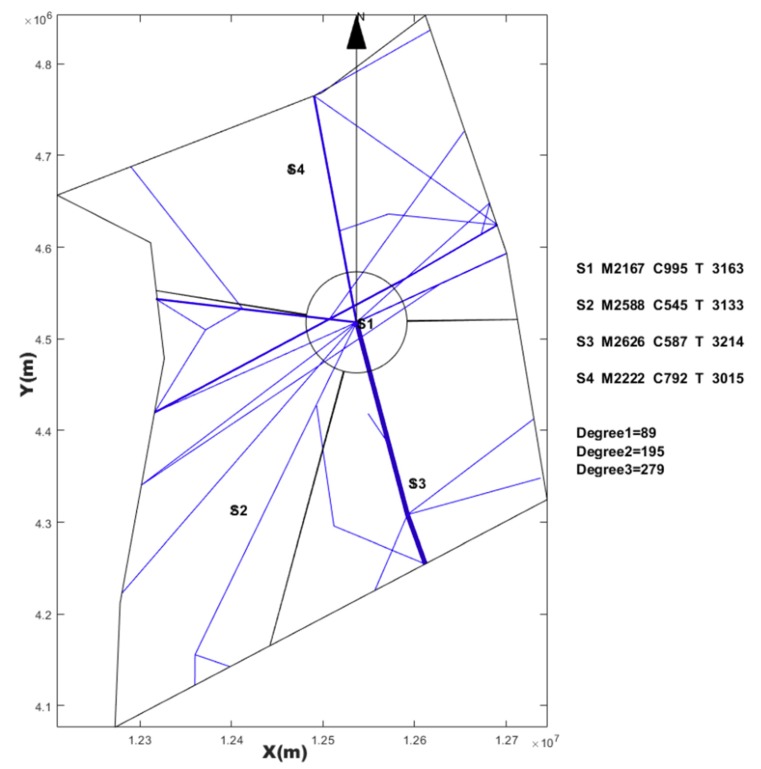
Hub-spoke optimizing partition using the MC-RC algorithm.

Figure 13 is a case of hub-spoke partition by optimizing the radius of the hub sector and bearing of the spoke using the MC-RC algorithm.

## 4. Results

We extracted one week’s data from Shanxi regional airspace. Since there were few flights between 20:00 p.m. and 7:00 a.m. in Shanxi regional airspace, we only counted the traffic flow data of each segment from 7:00 a.m. to 20:00 a.m. and took the average value to calculate the task load during the reconfiguration process. The parameters used in different algorithms are shown in Table 4.

### 4.1. SAA Test Result

Based on the three Voronoi diagrams, we used the SAA described in Section 3 to execute the automatic sector optimization calculation for the studying airspace. The results comparing are shown in Table 5.

**Table 5:** Comparing simulated annealing algorithm (SAA) results with three kinds of seeds generating method.

Seeds Generating Method	TL in Sector1 (s)	TL in Sector2 (s)	TL in Sector3 (s)	TL in Sector4 (s)	Std	TTL in Airspace (s)	Figure
Based on randomly	3316	2409	2443	2023	546.4	10,191	Figure 14
Based on every 50 km	2618	2459	2688	2890	178.8	10,655	Figure 15
Based on hexagonal	2383	3144	2493	2265	393.0	10,285	Figure 16
Current configuration	3018	2503	4025	3031	636.6	12,577	Figure 17

Note: TL is the abbreviation of task load. TTL is the abbreviation of total task load. The (s) is the abbreviation of seconds. Std is the abbreviation of standard deviation.

### 4.2. Post-Processing Result

The first purpose of post-processing was to smooth the boundary while keeping the task load balance and other constrains. The second purpose was to compare the results of post-processing with MC-CLFV and MC-RC. The results of post-processing graph are shown in Figure 18, Figure 19 and Figure 20.

Task loads after post-processing based on three kinds of seeds and task load evaluated based on current configuration are shown in Table 6.

### 4.3. Computational Efficiency Evaluation

The annealing parameters of the SAA algorithm are the initial temperature $T_{0}$ and termination temperature is $T_{min}$, and cooling rate of $T_{0}$ in every iteration is σ. According to the basic idea of the simulated annealing algorithm, the cycle times of outer loop could be $log_{\sigma }^{\frac{T_{min}}{T_{0}}}$, the cycle times of inner loop is set as m. Therefore, the calculation time of SAA could be expressed as:

(30) $$T_{SAA}=\;log_{\sigma }^{\frac{T_{min}}{T_{0}}}\cdot \;m\cdot T_{inner},$$

where $T_{inner}$ is the time when the inner loop is executed once. The $T_{inner}$ has a relationship with the calculation time of the monitor workload ($L_{R}\cdot L_{S})$, calculation time of the coordination workload ($L_{R}\cdot L_{S}\cdot L_{C1}\cdot L_{C2})$ and calculation time of the structural adjustment of sectors ($L_{C1}^{2})$. The $L_{R}$ is the time the program traverses all routes once. The $L_{S}$ is the time the program traverses all sectors. The $L_{C1}$ is the time the program traverses all Voronoi cells. The $L_{C2}$ is the time the program traverses all boundaries of Voronoi cells. The $T_{inner}$ could be expressed as:

(31) $$T_{inner}=L_{R}\cdot L_{S}+L_{R}\cdot L_{C1}\cdot L_{C2}\cdot L_{S}+L_{C1}^{2}.$$

Therefore, the final expression of the calculation time of SAA is:

(32) $$T_{SAA}=\;log_{\sigma }^{\frac{T_{min}}{T_{0}}}\cdot \;m\cdot (L_{R}\cdot L_{S}+L_{R}\cdot L_{C1}\cdot L_{C2}\cdot L_{S}+L_{C1}^{2}).$$

The calculation time of the SAA algorithm with different numbers of cells and sectors is shown in Figure 21. The calculation time of the SAA algorithm increased with the increase of the numbers of cells and sectors. When the number of cells was large, the difference of the computing time caused by the increase of the number of sectors became larger.

The cycle times of the MC-CLFV algorithm are a random constant ξ. The circulation time for one execution of the MC-CLFV algorithm consists of three major parts: Monitor the workload calculation time ($L_{R}\cdot L_{S})$, calculation the time of the structural adjustment of sectors ($L_{VF}\cdot L_{D\_min}\cdot L_{S})$ and calculation the time of the coordination workload calculation ($L_{R}\cdot L_{C1}\cdot L_{C2}\cdot L_{S})$. The $L_{VF}$ is very short, because it is the time that all flexible vertices are located. The $L_{D\_min}$ is the detection time for the minimum distance constraint (from the key intersection to sector boundary). The theoretic calculation time of the MC-CLFV algorithm could be expressed as the following:

(33) $$T_{MC-CLFV}=\xi \cdot \left(L_{R}+L_{R}\cdot L_{C1}\cdot L_{C2}+L_{VF}\cdot L_{D\_min}\right)\;\cdot L_{S}.$$

Although the MC-CLFV algorithm also consists of three major parts as the SAA algorithm, the factual runtime decreases dramatically because of a new sector adjustment method by introducing flexible vertices. The calculation time of the MC-CLFV algorithm increased with sector numbers as shown in Figure 22.

The reason for the increase of the calculation time is not only the increase of the number of sectors, but also the reduction of the area of each sector caused by the increase of the number of sectors, which makes it difficult to meet the minimum distance constraint between critical intersections and intersections. This leads to the continuous generation of infeasible sector boundaries, which increases the computational time. As shown in Figure 23, when the number of sectors was constant, the computational time increased with the minimum distance constraint, which proved our reasoning.

The theoretical calculation time of the MC-RC algorithm is similar to that of the MC-CLFV algorithm. Since it only needs to adjust the radius of the central circular sector and the angle of the rays that make up the other sectors, the reconstruction time is shorter. The theoretic circulation time of the MC-RC algorithm could be expressed as the following:

(34) $$T_{MC-CLFV}=\xi \cdot \left(L_{R}+L_{R}\cdot L_{C1}\cdot L_{C2}+L_{G}\cdot L_{D\_min}\right)\;\cdot L_{S}.$$

The $L_{G}$ is the reconstruction time of sectors for just once.

When the number of sectors is equal to 4, the average calculation time of the MC-RC algorithm is very short. The worst case consumes only about 140 s. However, when the number of sectors is greater than 4, the program will hardly terminate without changing the termination condition (standard deviation of sector workload). In order to test the computational speed of more than four sectors, we increased the standard deviation from 100 to 400. The worst case could consume about 1700 s. It could be inferred that the MC-RC algorithm was not suitable for constructing more than four sectors in Taiyuan airspace.

## 5. Discussion

From the result of SAA in Table 5, the sector generated by SAA method was more balanced than the current sector workload, and the total workload was smaller. We also found that the solution space of SAA varied with the seeds generating method and the number of seeds. Three Voronoi diagram generation methods, combined with the number of variable seed points, improved the reliability of airspace design meeting the required performance standards. This revealed that there were defects in some previous literatures, which only depended on one method to discretize the airspace. Although simulated annealing is a very mature algorithm, it must be pointed out that adding the re-entry sector penalty and short dwell time penalty to the objective function will have an impact on the optimization search. If the weight of these two penalties is too large, the algorithm will be limited to local optimization, and if the settings are too small, there will be a re-entry sector and short dwell. However, because the simulated annealing algorithm accepts infeasible solutions by itself with a certain probability, it allows the re-entry sector and short residence to occur in the exploration process, which makes it possible to explore the global optimization solution, and makes it possible for the region growth method to produce sectors that meet the performance requirements. Using SAA to find a good initial solution lays a foundation for a warm start in post-processing.

**Figure 14:**
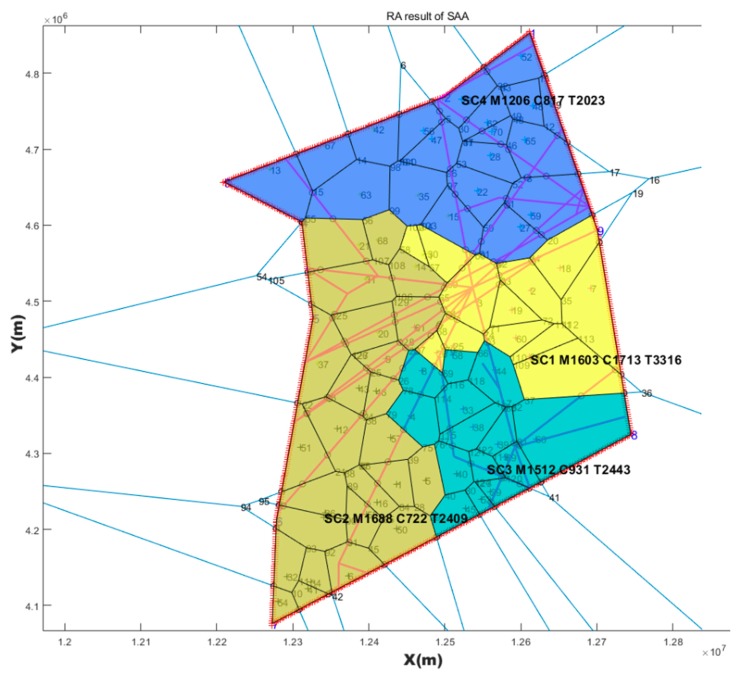
Reconfiguration results based on random seeds with *SAA*.

**Figure 15:**
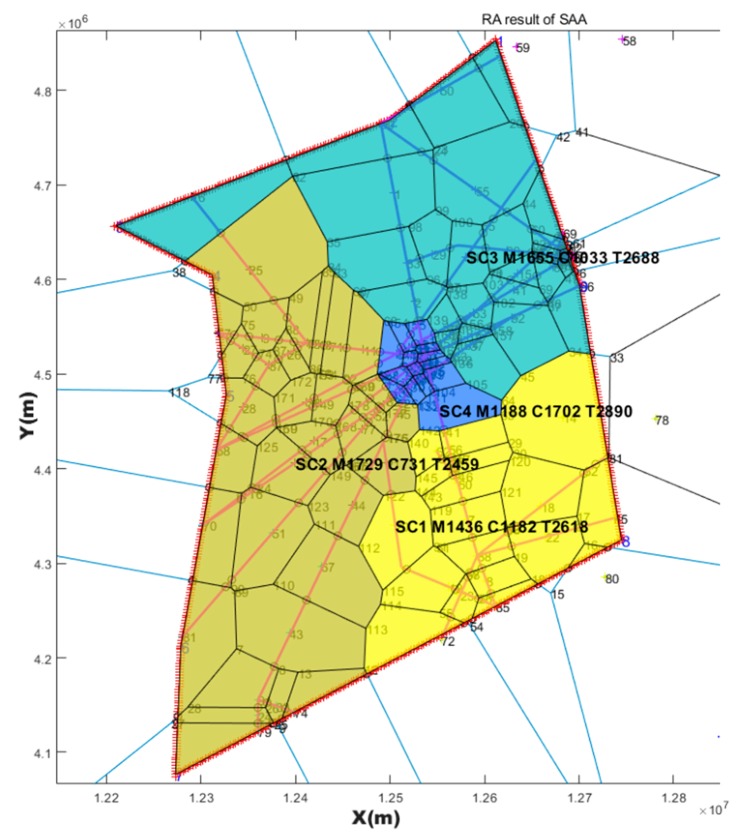
Reconfiguration results based on seeds every 50 km with *SAA*.

**Figure 16:**
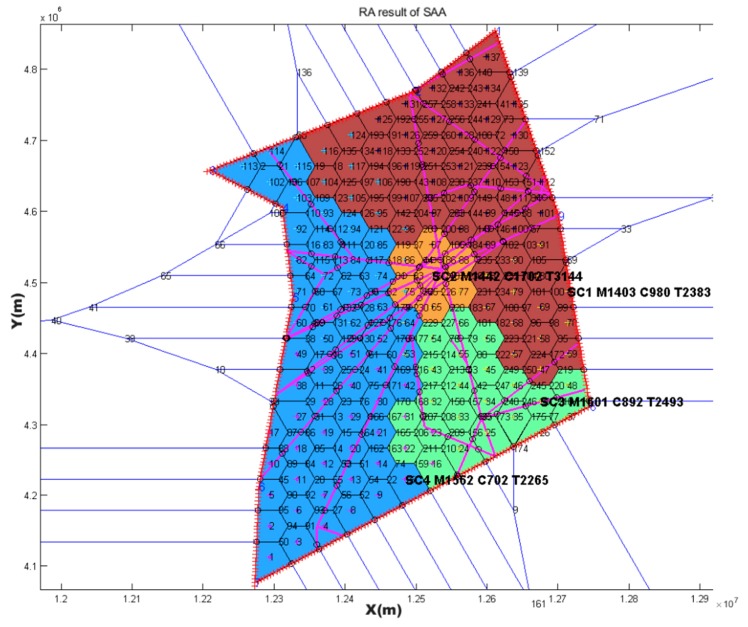
Reconfiguration results based on hexagonal seeds with *SAA*.

**Figure 18:**
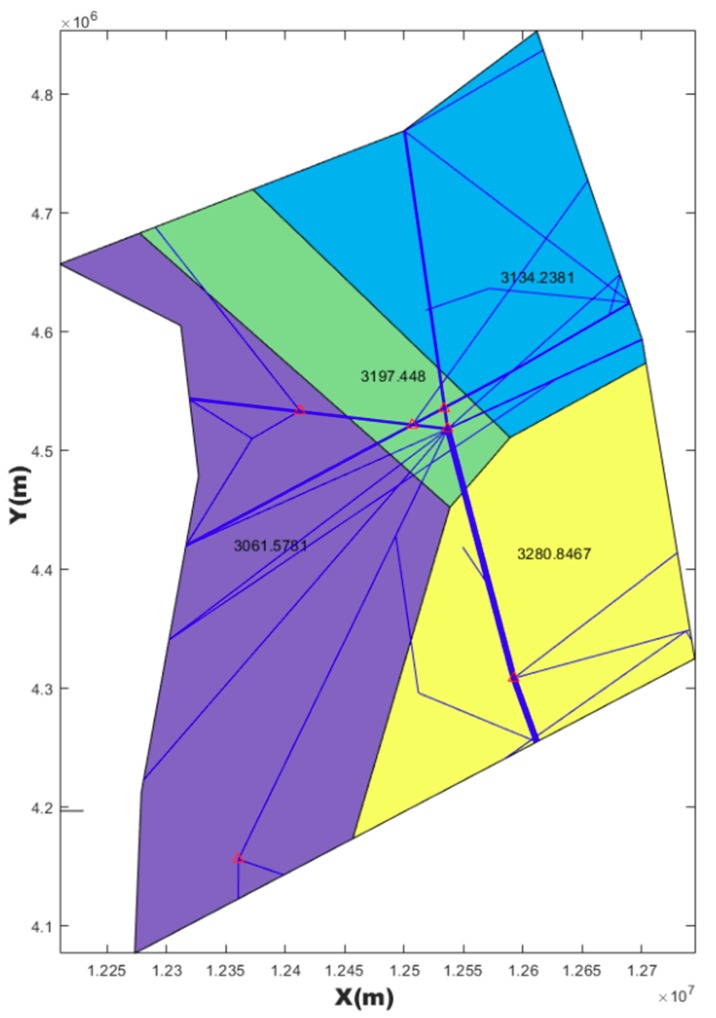
Post-processing results of Figure 14 with MC-CLFV.

**Figure 19:**
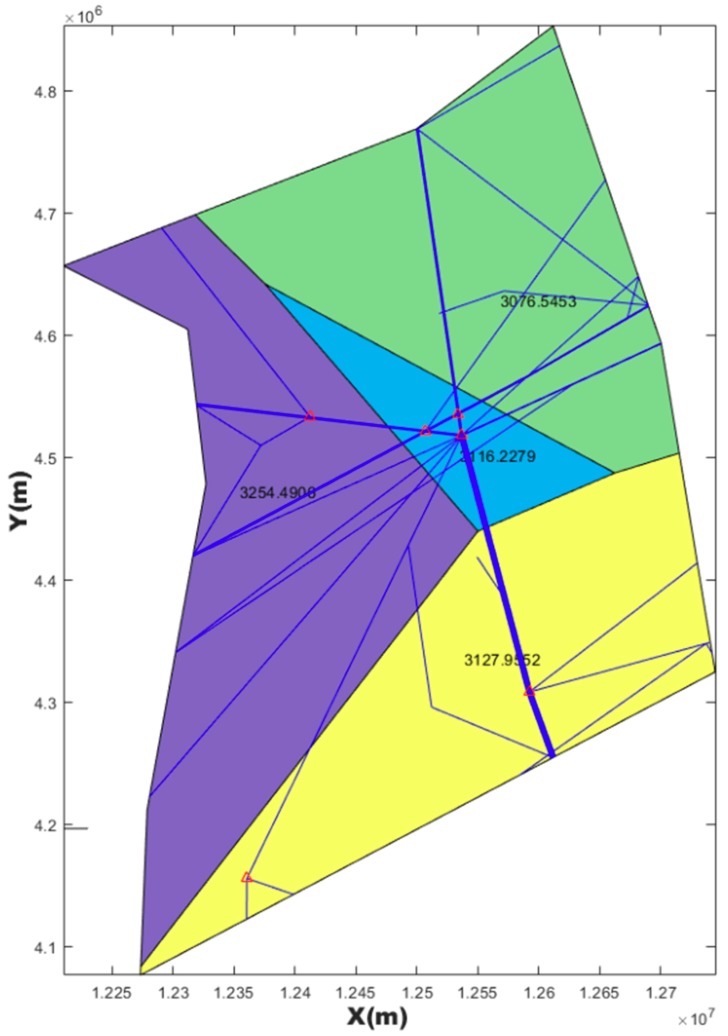
Post-processing results of Figure 15 with MC-CLFV.

**Figure 20:**
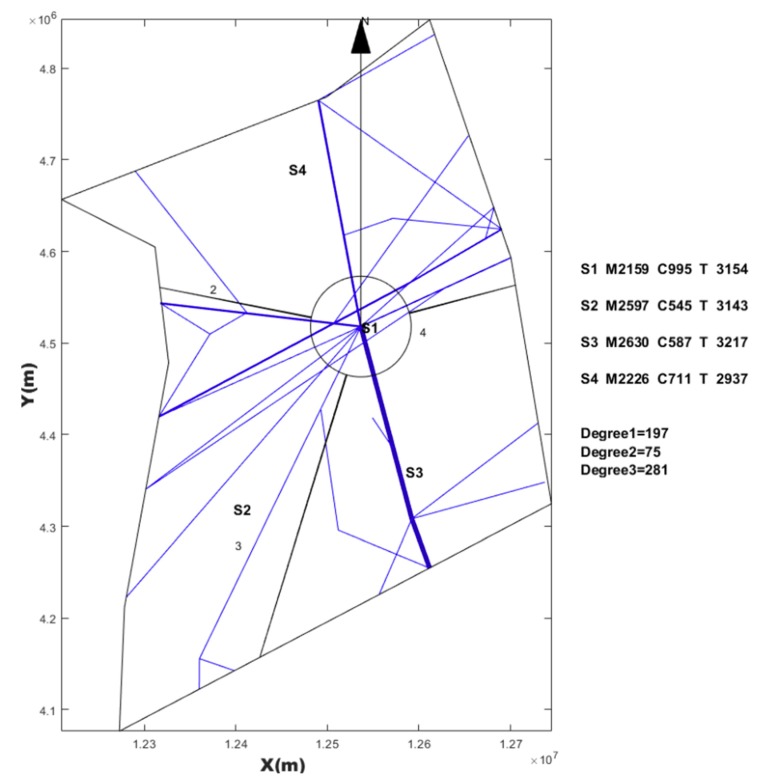
Post-processing results of Figure 16 with MC-RC.

**Figure 21:**
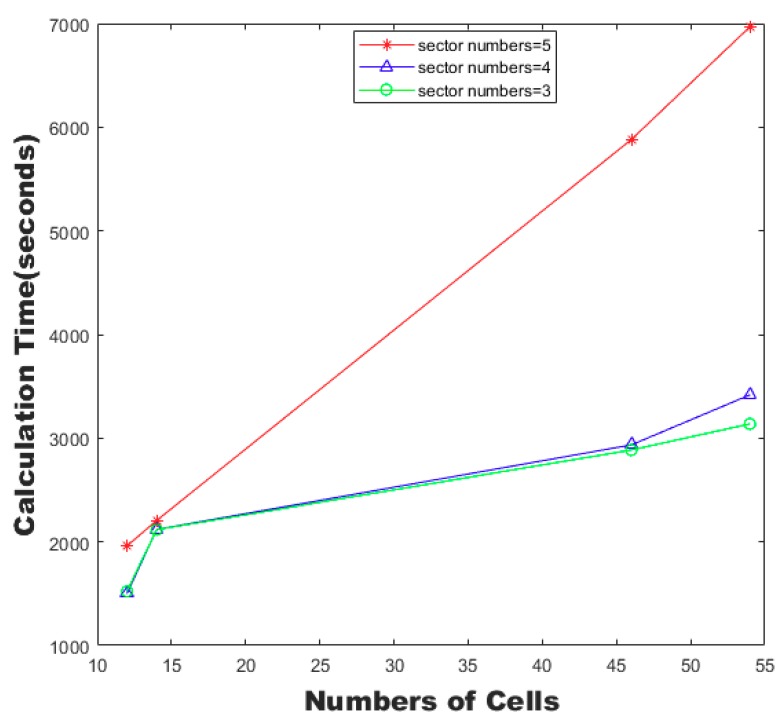
Calculation time of the SAA algorithm with different numbers of cells and sectors.

**Figure 22:**
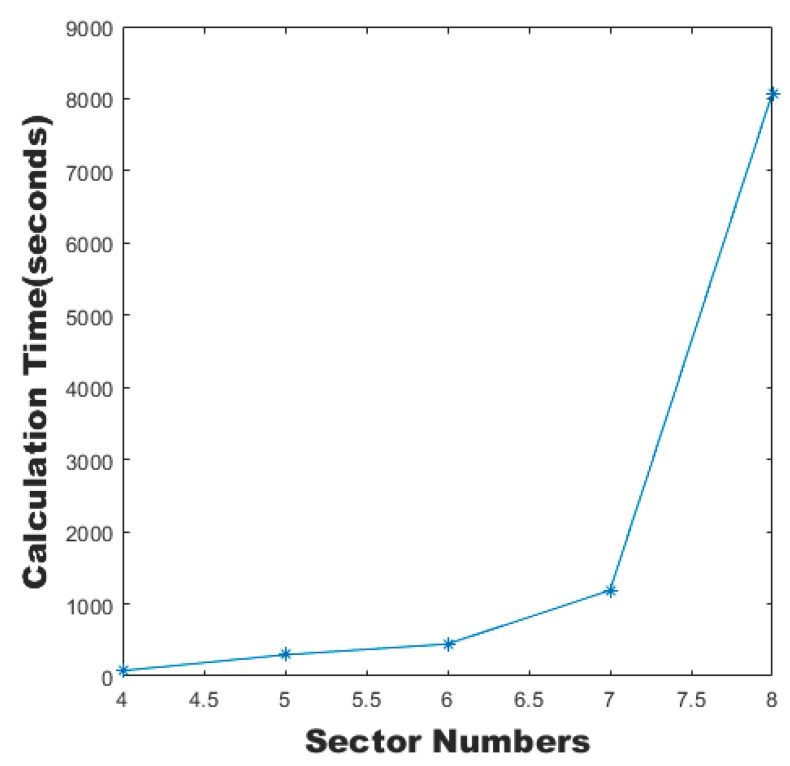
Calculation time of the MC-CLFV algorithm with different sector numbers.

**Figure 23:**
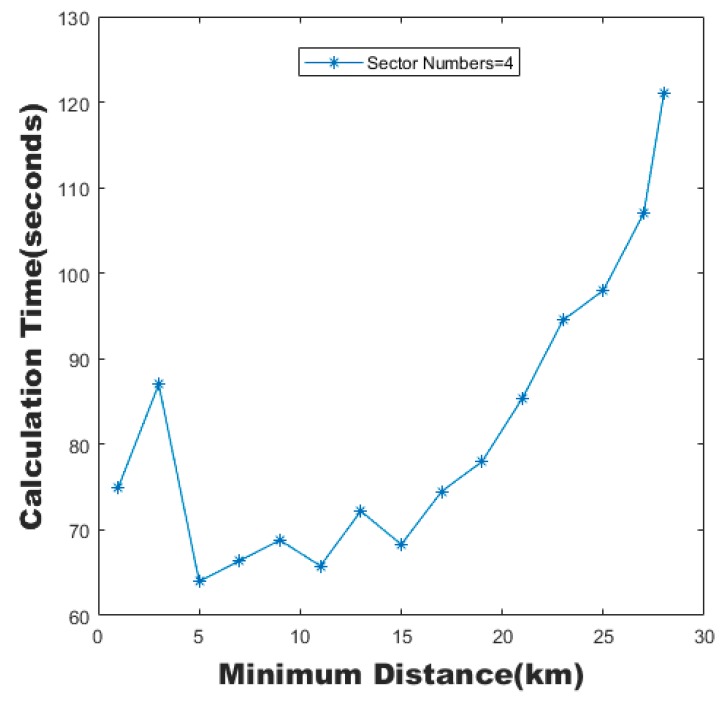
Calculation time of the MC-CLFV algorithm with different minimum distances.

However, irregular sector boundaries in Figure 14, Figure 15 and Figure 16 were not satisfactory. Any method based on discrete airspace block reorganization would have such problems as zigzag, c-shaped, trapezoidal and so on. Therefore, effective post-processing is very important. The result in Table 6 shows that the two proposed post-processing methods could achieve a more balanced partitioning scheme than the existing sector. The MC-CLFV algorithm could get more balance configuration than MC-RC, but the MC-RC algorithm could get smaller total task load. The choice between the two performance criteria, the minimum total workload and the balanced task load of each sector, seems to be contradictory. Of course, we expected the minimum total task load and the balanced task load of each sector at the same time. However, when we cannot have both of them, how do we choose? Answering this question needs to be traced back to the purpose of AS. The fundamental purpose of AS is to increase traffic throughput. Especially when the current throughput of airspace is mainly limited by the task load of controllers, the purpose of AS is to reduce the average task load of controllers, regardless of whether the number of sectors remains unchanged or by increasing the number of sectors. When the number of sectors and the demand of controllers remain unchanged, a more balanced scheme is preferred for the fundamental purpose of AS. Since only in this way, each sector has more buffers to cope with fluctuating traffic flow and traffic growth. Therefore, we concluded that the MC-CLFV algorithm was superior to the MC-RC algorithm in Shanxi airspace for increasing traffic throughput. However, compared with Figure 20 and Figure 18 and Figure 19, it was found that there were no narrow blocks in the sector generated by MC-RC, and MC-RC seemed to show some advantages when it was necessary to utilize the space of narrow blocks. It must be mentioned that in order to evaluate the computing time of the MC-RC algorithm, we relaxed the sector workload balancing constraints. When the number of sectors is more than 4, there may be no feasible solution without relaxing the sector workload balance constraints. This means that the MC-RC algorithm is not as applicable as MC-CLFV algorithm. We believed that any algorithm has its use value in an appropriate environment.

Comparing the results of Table 5 and Table 6, we could find that although the sector generated by the SAA algorithm had irregular boundaries, the total task load was smaller than that after irregular boundaries were smoothed, which indicates that appropriately allowing some non-convex boundaries was a measure to reduce the total workload, and blindly pursuing convex boundaries would increase the total workload.

## 6. Conclusions

Partitioning the airspace into optimal sectors, even 2D, is still a leading or forefront research issue. It is a multi-objective optimization problem, and the contradiction between multiple objectives or constraints makes the problem complicated. Many technologies and methods, such as Voronoi diagrams, k-mean clustering, graphic reshaping technology, graph coding technology, visualization, task load calculation, the simulated annealing algorithm (SAA) and variable neighborhood search strategies, post-processing of sector boundary based on the Monte Carlo method and morphology, were synthetically used to realize automatic AS in this paper. The modeling approach and algorithm solution presented in this paper were tested and compared to existing sectorization. The provided results demonstrated that the proposed sector design method framework was able to provide very satisfying sectorization with regards to sector load balancing, as well as to total task load minimization, minimum distance to boundary, the number of re-entering and short-dwell criteria.

The graph cutting algorithms proposed by us had two theoretical contributions. The first was to eliminate the inherent problem of irregular sector boundaries generated by region growth algorithm. Second, because of the results of the previous region growing algorithm as a hint, how many sectors and the number of flexible vertices in the graph cutting algorithm became known parameters. Therefore, the AS problem became the problem of determining the location of flexible vertices. This greatly simplifies the complexity of the graph cutting algorithm.

The proposed method framework of AS and software could provide reliable assistant design and analysis tools for airspace planners to design airspace, improve the reliability and efficiency of design and reduce the burden of airspace planners while guaranteeing the key performance indicators in the process of AS. In addition, this laid the foundation for reconstructing airspace with a more intelligent method. The presented methods when appropriately adapted, could be applied in another partitionable system, e.g., in land partitioning, power network partitioning, communication network partitioning and other transport sectors, e.g., in technological processes scheduling, rescue actions, crisis management, etc.

We did not claim that we had completed the full-automatic design of the airspace. Since airspace design is a complicated process, we had only solved the problem that the initial airspace design was almost completed manually in China in the past. This scheme has to be further simulated and evaluated by SIMMOD, AIRTOP and other tools [78]. Finally, it has to be evaluated by human in the loop before it can be implemented [6]. Some modifications will even be made based on feedback in a field test. This reflects the fact that there is a huge gap for improvement in the current task load model and airspace sectorization model. It is also a great opportunity and challenge for researchers to automate the whole process of airspace design.

In general, the proposed method framework not only satisfied a variety of key performance indicators, but also eliminated the irregular sector shape generated by the regional growth method, which laid the foundation for improving the reliability and acceptability of the airspace design and enriches the automatic airspace reconstruction method.

## Figures and Tables

**Figure 17:**
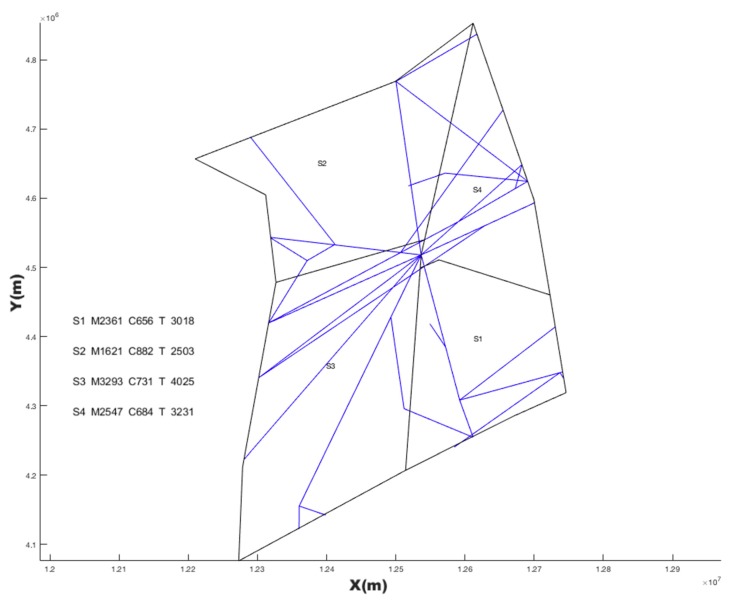
Current configuration and task load.

**Table 1:** Examples of the route network and daily traffic volume.

Route	$\mathrm{Starting}\;\mathrm{Point}\;\mathrm{Coordinates}\;(\varphi_{1},\lambda_{1})$		$\mathrm{End}-\mathrm{Point}\;\mathrm{Coordinates}\;(\varphi_{2},\lambda_{2})$		Starting Point	End Point	Daily Traffic Volume *f* (Flights/Day)
B208(N)	39°29.5′	112 12.1	37°45.0′	112°37.1′	TODAM	TYN	146
B215(E)	37°45.0′	112 37.1	38°17.3′	114°05.9′	TYN	ISGOD	90
G212(W)	37°45.0′	112 37.1	36°28.4′	110°30.6′	TYN	OKVUM	17

**Table 2:** First type of representation of solution z.

VC	1	2	3	4	5	6	7	8	9	10
Sector 1	1	1	1	0	0	0	0	0	0	0
Sector 2	0	0	0	1	1	1	0	0	0	0
Sector 3	0	0	0	0	0	0	1	1	1	1

**Table 3:** Second type of representation of solution x.

VC	1	2	3	4	5	6	7	8	9	10
Sector	1	1	1	2	2	2	3	3	3	3

**Table 4:** Parameters used in the algorithm.

Parameters	Value	SAA	MC-CLFV	MC-RC
Task load imbalance	$\alpha_{1}$ = 4000	√	√	√
Total coordinating task load	$\alpha_{2}$ = 200	√	√	√
Cost of a short dwell time	$\alpha_{3}$ = 5	√	√	√
Cost of reentering the same sector	$\alpha_{4}$ = 1500	√	√	√
Initial temperature	$T_{0}$ = 1e+3	√		
Termination temperature	$T_{min}$ = 1e–3	√		
Cooling rate	σ = 0.8	√		
Radius decreasing rate in every iteration	$\sigma^{1\;}$ = 0.96		√	
Monitoring task load per 10 min	$\alpha_{mot}$ = 22/600 (*s*/*s*)	√	√	√
Coordination task load required for each handover	$\alpha_{cod}$ = 9 (s/flight)	√	√	√
Permissible minimum of dwell time	$t_{dw}^{min}$ = 240 (s)	√	√	√
The minimum deviation from the average of the total task load	$\beta_{1}$ = 0.5	√	√	√
The design redundancy parameter	$\beta_{2}$ = 0.95	√	√	√
The maximum acceptable standard deviation	τ = 100		√	√
The maximum acceptable task load in each sector	$wl_{max}\;$ = 3420 (s)	√	√	√

**Table 6:** Comparison of results between the MC-CLFV algorithm and MC-RC algorithm.

Algorithm	Original Configuration	After Post-Processing	TL of S1 (s)	TL of S2 (s)	TL of S3 (s)	TL of S4 (s)	Std	TTL in Airspace (s)
MC-CLFV	Figure 14	Figure 18	3061.6	3197.5	3134.2	3280.9	93.2	12,674
MC-CLFV	Figure 15	Figure 19	3254.5	3076.6	3116.2	3128.0	77.0	12,575
MC-RC	Figure 16	Figure 20	3154	3143	3217	2937	121	12,451
Current configuration	Figure 17		3018	2503	4025	3231	632.6	12,777

Note: The abbreviations in this table have the same meaning as in Table 5.
